# Evolution of clinical benefit in cancer trials: comparing pivotal approval data with updated efficacy

**DOI:** 10.3389/fpubh.2026.1876451

**Published:** 2026-07-03

**Authors:** Dongdong Zhang, Fang Fang Cai, Guangpeng Chen, Liping Zhang

**Affiliations:** 1 Department of Hematology, Hebei Petro China Central Hospital, Langfang, China; 2 Department of Public Health, Fudan University, Shanghai, China; 3 Department of Anesthesiology, Hebei Petro China Central Hospital, Langfang, China

**Keywords:** clinical trial, evolution of efficacy, FDA approval, oncology drugs, restricted mean survival time

## Abstract

**Background:**

Many oncology drugs are approved by the Food and Drug Administration (FDA) based on interim analyses from randomized controlled trials (RCTs) using immature efficacy data. Although expedited approvals expedite access, they create uncertainty about the durability of benefit and long-term efficacy. We aimed to systematically compare initial and updated results from RCTs supporting FDA oncology approvals, evaluating longitudinal changes in relative efficacy.

**Methods:**

All phase II–III RCTs supporting FDA oncology drug approvals from 2006–2025 that reported both initial and updated results were included. Relative efficacy (overall survival [OS] and progression-/event-free survival [PFS/EFS]) between groups was quantified using restricted mean survival time (RMST) ratio, with changes over time expressed as Ratio of updated-to-initial RMST ratio (RR). Meta-analyses and meta-regressions were used to estimate pooled effects and identify trial-level factors. Multiple sensitivity analyses, including those based on RMST difference (RMSD), were conducted to assess robustness.

**Results:**

A total of 165 RCTs were included. For OS, mean RR was 1.052 (95% CI, 1.029–1.063) and meta-analytic RR 1.023 (1.015–1.031, *I*^2^ = 15.5%); for PFS/EFS, mean RR was 1.131 (1.102–1.153) and meta-analytic RR 1.099 (1.078–1.121, *I*^2^ = 69.2%), indicating significant relative survival improvement for experimental arms as follow-up matured. 84.7% of OS and 92.5% of PFS/EFS comparisons showed increased RRs. Sensitivity analyses showed normalized RMSDs of 0.072 months/year (OS) and 0.156 months/year (PFS/EFS) in experimental arms. Shorter initial follow-up and less mature control-arm data were associated with greater relative efficacy gains of experimental treatments.

**Conclusions:**

With extended follow-up, most FDA-approved oncology drugs with available updated data showed more durable survival benefits. Given the inherent selection of trials with publicly available updates and reconstructable survival curves, these findings should not be interpreted as a global validation of FDA approval decisions, but they underscore the need for dynamic, evidence-maturity-based oversight with ongoing reassessment and strengthened post-marketing surveillance.

## Introduction

In contemporary United States (US) regulatory practice, many oncology indications are approved based on interim analyses from ongoing randomized clinical trials (RCT) and surrogate endpoints such as progression-free survival (PFS) or response rate, while overall survival (OS) data remain immature (1). Expedited pathways and reliance on early, information-sparse evidence accelerate patient access but introduce substantial uncertainty regarding the durability of benefit and long-term efficacy (2). Comparing the evidence available at approval with subsequent updates from the same RCTs is therefore crucial to determine whether survival advantages persist or expand over time.

Evaluating these changes requires integrating evidence from time-to-event endpoints (OS, PFS), with careful attention to methods quantifying treatment effects. As underscored by Soon et al. (3), changes in treatment effects can be quantified using the ratio of updated to initial estimates from interim and subsequent analyses. For time-to-event endpoints, hazard ratio (HR)-based inference relies on the proportional hazards (PH) assumption, which is often violated in modern oncology trials due to delayed treatment effects, crossover, evolving post-progression therapies, and time-varying hazards (4). A comparative assessment of oncology RCTs found that about one quarter of trials exhibited non-proportional hazards (5). Additionally, RCTs that met the PH assumption at initial analysis may no longer do so with extended follow-up. In this context, HRs become difficult to interpret and may misrepresent the evolution of treatment benefit over time (5).

Restricted mean survival time (RMST), defined as the area under the survival curve up to a prespecified truncation time (6), provides a robust alternative or complement to HRs in this setting. RMST does not rely on the PH assumption, remains valid under time-varying hazards, and directly quantifies average survival within a fixed horizon (7); clinically, it offers an intuitive measure of absolute and relative survival gains readily interpretable by patients and clinicians (8). These features make RMST particularly suitable for evaluating how treatment effects evolve as oncology trials mature from approval-time analyses to later updates, especially for novel therapies such as immunotherapies, innovative targeted therapies, and combination regimens with delayed but durable benefits (9, 10).

Recent evaluations have highlighted persistent uncertainty in the evidentiary foundation of the US Food and Drug Administration (FDA) oncology drug approvals. Gloy et al. (11) reported that nearly half of cancer drug indications approved from 2000–2020 lacked randomized evidence and were often based on surrogate endpoints. Similarly, Liu et al. (2) analyzed oncology drugs granted accelerated approval and found that most did not demonstrate OS improvement within 5 years, with many confirmatory studies still relying on surrogate rather than clinical endpoints. Another recent study including RCTs published in four major general medical journals showed time-dependent changes in efficacy estimates but relied solely on HRs, which may bias results when PH assumption is violated (5). Collectively, prior evaluations of oncology drug approvals by FDA have largely provided cross-sectional assessments of evidence at the time of approval (2, 12–14), while most analyses have been confined to specific tumor types or accelerated approvals and rarely examined how efficacy and safety evolve with longer follow-up. In addition, interpretation of efficacy in these work has relied heavily on HRs, yet the growing occurrence of non-PH can bias these estimates (3). To address these gaps, we systematically reviewed RCTs supporting FDA oncology approvals, quantified changes in relative efficacy between innovative therapies and controls, and explored trial-level factors associated with these variations, using PH-independent metrics to capture how therapeutic benefit evolves over time and to inform evidence-based clinical and regulatorydecision-making.

## Method

**Figure 1:**
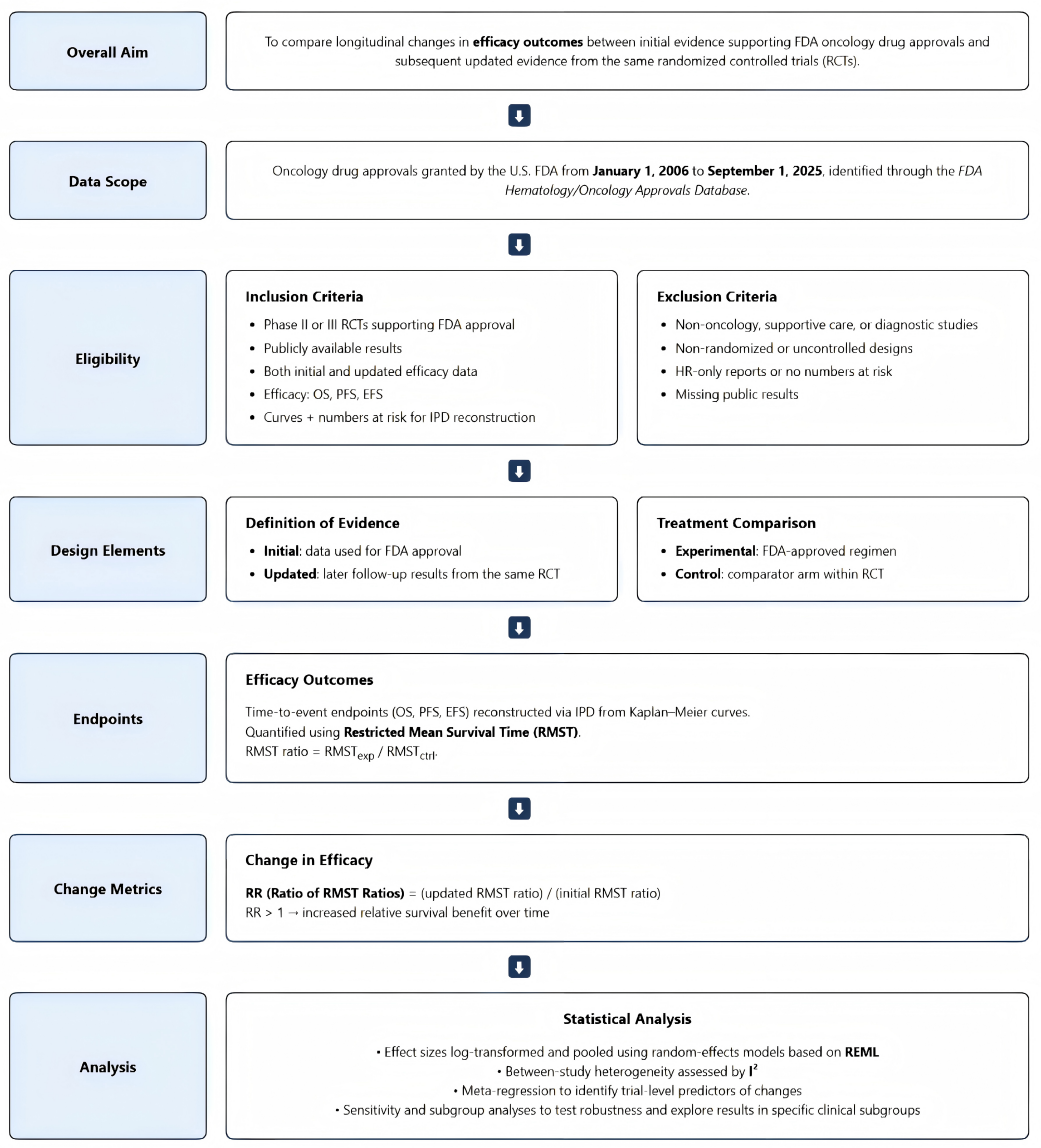
Study process flowchart. RR, ratio of the updated to initial restricted mean survival time (RMST) ratio; ROR, ratio of updated to initial odds ratios; OS, overall survival; PFS/EFS, progression-free/event-free survival.

Overall study process is outlined in Figure 1.

### Sample identification

We identified all oncology drug approvals granted by the US FDA between January 1, 2006, and September 1, 2025, using FDA Hematology/Oncology Approvals Database (https://www.fda.gov/drugs/drug-approvals-and-databases/resources-information-approved-drugs). Eligible studies were phase II or III RCTs supporting these approvals. To assess longitudinal changes in efficacy, each included RCT was required to report both initial evidence (data considered by the FDA at approval) and updated evidence from later publications. Efficacy outcomes included OS, PFS, or disease-free, relapse-free, or event-free survival (collectively referred to as EFS). To reduce potential bias related to non-PH risks, survival outcomes were analyzed using RMST methods. Trials that reported only HRs without survival curves (essential for individual patient data [IPD] reconstruction), or survival curves without numbers at risk (essential for accurate IPD reconstruction), were excluded. Approvals for non-oncology, supportive care, or diagnostic indications, as well as non-randomized or uncontrolled studies, were also excluded.

Publication records of supporting RCTs were identified in MEDLINE and ClinicalTrials.gov using NCT numbers, drug names, and indications. When multiple updated datasets were available, only the most recent update was included as the updated evidence. Two reviewers (DZ and FC) independently screened all records according to predefined inclusion and exclusion criteria, with disagreements resolved through discussion until consensus was reached.

### Data extraction

Four authors (GC, PZ, DZ, and FC) independently performed data extraction. For each trial, we collected data on study design (trial phase, blinding, and crossover allowance), FDA approval date of indication, treatment comparisons, cancer indication, sample size, follow-up duration, initial and updated survival outcomes and corresponding information fraction (IF, defined as proportion of observed events relative to total number of events specified in original power calculation) (15). For survival outcomes, Kaplan–Meier curves were digitized using DigitizeIt (version 2.0.4, Bormisoft, Braunschweig, Germany), and IPD were reconstructed using the method described by Guyot et al. (16) Accuracy of IPD reconstruction, evaluated by comparing the estimated number-at-risk and survival rate at each time point with those reported in original RCTs, was required to meet all of the following criteria: root mean square error < 0.01, mean absolute error < 0.005, maximum absolute error < 0.01, and a large *p*-value (>0.9) in Kolmogorov–Smirnov test. These thresholds were set much more stringently than those used in previous study (17) to ensure higher reconstruction accuracy. Additionally, reconstructed IPD were further used to generate Kaplan–Meier curves, which were independently verified by two reviewers (DZ and FC) for consistency with the published curves.

### Assessment of changes in relative treatment effects

In our study, the primary efficacy endpoints considered were OS and PFS/EFS (18). In each RCT, innovative therapy that received FDA approval was defined as the experimental arm, whereas the comparator regimen was defined as the control arm. RMST ratio was calculated to compare relative survival between experimental and control groups (3). For each trial, RMST was estimated as the area under the Kaplan-Meier survival curve from time zero to *t*_*max*_ (τ, defined as the shorter of the maximum observed follow-up times in two arms). RMST ratio (RMST_exp_/RMST_con_) greater than 1 indicated a survival advantage for the experimental arm (19).


$$\begin{array}{l} RMST=\int_{0}^{t_{\max}}S\left(t\right)dt,\;RMST\;ratio=\frac{RMST\;of\;Experimental\;group\;}{RMST\;of\;Control\;group} \end{array}$$

To evaluate changes in relative treatment effect over time, both the initial and updated RMST ratios were calculated, and their ratio (updated-to-initial RMST ratio, RR) was derived (20). An RR > 1 indicated that relative survival advantage of experimental treatment increased in updated report (5).


$$\begin{array}{l} RR\;\left(ratiobetweenRMST\;ratio\right)=\;\frac{RMST\;ratio\;in\;updated\;data}{RMST\;ratio\;in\;initial\;data} \end{array}$$

Because τ inevitably differs between initial (τ1) and updated (τ_2_) analyses, we did not compare absolute RMSTs across time points; instead, the within-analysis between-arm RMST ratio was used, and the updated-to-initial ratio (RR) was taken as a scale-free metric largely independent of the absolute length of follow-up. To further address potential bias from sparse tail data and shifting truncation horizons, RMSTs were recalculated at 95% and 90% of τ, and absolute survival gains were normalized to a per-year scale (nRMSD) for cross-time comparability.

### Statistical analysis

To assess whether relative efficacy between treatments changed after data updates, the logarithms of metrics (ROR) were used as effect sizes, with corresponding standard errors estimated using the delta method based on reported standard errors before and after data updates (5). Random-effects meta-analyses were then conducted using an inverse-variance-weighted approach, with pooled effect sizes estimated via restricted maximum likelihood method (REML) method. Between-study heterogeneity was quantified using the I (2) statistic (21).

For pooled estimates of RMST ratios in initial and updated evidence, effect sizes were meta-analyzed on the log scale using an inverse-variance weighted random-effects model (REML method) (21). A Wald-type *Z* test, based on difference in log-transformed RMST ratios and their standard errors, was used to assess whether updated RMST ratio was significantly different from initial estimate for each trial (22).

Meta-regression analyses were performed to identify trial-level factors associated with the magnitude of treatment effects and to explain potential sources of heterogeneity. Dependent variable was the log-transformed metrics. Covariates were prespecified a priori based on clinical relevance and data availability, and summarized in Supplementary Table S1. To prevent overfitting, we followed the rule of at least 10 studies per covariate. Each available covariate was first analyzed using univariable meta-regression, and those with *p* < 0.10 were included in a multivariable model. Multicollinearity among covariates was assessed using variance inflation factors (>5 indicating potential collinearity) (23).

The PH assumption was formally evaluated for each treatment comparison, separately for OS and PFS/EFS in both initial and updated datasets, using the Schoenfeld residuals method (5) implemented via the R survival package. A two-sided *p*-value < 0.05 was prespecified as evidence of PH violation, with residual plots inspected as visual confirmation.

### Subgroup analyses and sensitivity analyses

Subgroup analyses were conducted to examine whether changes in relative treatment effects varied according to trial-level characteristics. The following subgroups were prespecified a priori based on clinical and methodological considerations: PH status, year of FDA approval, cancer type, treatment line, intervention comparison, trial phase, blinding, crossover allowance, metastatic setting, trial sample size, 5-year survival rate of the underlying cancer, follow-up duration, right-censoring rate of survival curves, and IF. The subgroups for each outcome, along with the rationale for their selection, are summarized in Supplementary Table S2.

To assess the robustness of the primary findings, several sensitivity analyses were performed. First, to complement relative survival estimates, absolute survival gain between experimental and control arms was quantified using Restricted Mean Survival Time Difference (RMSD) (24), then normalized to an annualized value (nRMSD, RMSD divided by truncation time [in years]). Change in absolute survival benefit over time was evaluated as difference between updated and initial nRMSD values. Second, to minimize potential bias in RMST estimation due to sparse tail data, RMSTs were recalculated using truncated times corresponding to 95% and 90% of truncation time (τ).

## Results

A total of 524 records were identified through systematic searches, of which 329 RCTs met the eligibility criteria. After excluding 164 RCTs without subsequent updated outcomes, 165 RCTs with updated data were included in the final analysis. Characteristics of all included trials are summarized in Supplementary Table S3, and the study selection process is illustrated in Supplementary Figure S1. For RCTs included in OS analysis (details in Supplementary Table S4), eighty-nine studies evaluated first-line therapy, 114 focused on metastatic disease, and most were phase III (97%). Blinding was implemented in 51 studies, while crossover was permitted in 59 trials. Across tumor types, lung (*n* = 25), leukemia (*n* = 18), breast (*n* = 18), prostate (*n* = 12), kidney (*n* = 12), and melanoma (*n* = 10) cancers were most common. The most frequent treatment comparisons were immuno-chemotherapy vs. chemotherapy (*n* = 18), immunotherapy vs. chemotherapy (*n* = 13), targeted therapy vs. targeted therapy (*n* = 16), targeted-chemotherapy vs. chemotherapy (*n* = 11), and targeted vs. supportive care (or placebo; *n* = 10). Subgroup characteristics for other endpoints (PFS/EFS) are summarized in Supplementary Table S5. The distribution of trial-specific τ values is provided in Supplementary Tables S6, S7.

### Evolution of OS from Initial to updated analyses

A total of 150 paired comparisons were included in the OS analysis. Median maximum follow-up increased from 30.3 months (IQR, 22.5–40.5) in initial reports to 53.6 months (39.8–71.6) in updated analyses. Median censoring rates declined from 0.58 (IQR, 0.33–0.76) to 0.34 (0.19–0.56) in experimental groups and from 0.43 (0.23–0.66) to 0.25 (0.10–0.46) in control groups. PH assumption was met in 110 comparisons and violated in 40. More details are in Table 1.

**Table 1:** Summary of relative survival differences between FDA-approved innovative therapies and control groups before and after data updates.

Group	Comparisons with increased RMST ratios, *n* (%)		Comparisons with significantly increased RMST ratios, *n* (%)		Pooled initial RMST ratio (95% CI)		Pooled updated RMST ratio (95% CI)	
	OS	PFS/EFS	OS	PFS/EFS	OS	PFS/EFS	OS	PFS/EFS
All comparisons	123(82%)	111(93%)	7(5%)	24(20%)	1.08(1.07–1.10)	1.28(1.24–1.31)	1.13(1.11–1.14)	1.42(1.37–1.48)
FDA approve year								
Pre-2015	24(83%)	15(75%)	3(10%)	2(10%)	1.09(1.07–1.11)	1.33(1.24–1.42)	1.13(1.10–1.17)	1.43(1.32–1.55)
2016–2020	66(88%)	60(97%)	2(3%)	14(23%)	1.08(1.07–1.11)	1.26(1.21–1.31)	1.13(1.10–1.15)	1.42(1.35–1.49)
Post-2020	33(72%)	36(95%)	2(4%)	8(21%)	1.09(1.06–1.11)	1.28(1.21–1.35)	1.13(1.09–1.16)	1.43(1.32–1.55)
PH assumption								
PH	88(80%)	55(95%)	6(5%)	15(26%)	1.07(1.06–1.08)	1.26(1.21–1.31)	1.11(1.09–1.12)	1.40(1.33–1.48)
Non-PH	35(88%)	56(90%)	1(3%)	9(15%)	1.12(1.09–1.15)	1.29(1.24–1.34)	1.19(1.14–1.23)	1.45(1.37–1.53)
Treatment line								
First-line	76(85%)	71(96%)	5(6%)	17(23%)	1.07(1.6–1.09)	1.22(1.18–1.26)	1.12(1.10–1.14)	1.36(1.30–1.43)
Subsequent-line	47(77%)	40(87%)	2(3%)	7(15%)	1.10(1.08–1.13)	1.38(1.31–1.45)	1.14(1.11–1.17)	1.53(1.45–1.62)
Treatment comparison								
Immuno-chemotherapy vs. chemotherapy	17(94%)	12(100%)	1(6%)	2(17%)	1.09(1.07–1.12)	1.27(1.18–1.35)	1.15(1.11–1.19)	1.40(1.26–1.56)
Immuno-targeted vs. targeted	8(100%)	8(100%)	1(13%)	2(25%)	1.06(1.04–1.09)	1.27(1.18–1.38)	1.10(1.07–1.13)	1.41(1.28–1.55)
Immunotherapy vs. chemotherapy	13(100%)	11(100%)	0(0%)	2(18%)	1.17(1.14–1.21)	1.24(1.12–1.37)	1.26(1.21–1.31)	1.47(1.26–1.71)
Immunotherapy vs. supportive care/Placebo	4(50%)	11(100%)	0(0%)	0(0%)	1.07(1.02–1.11)	1.24(1.16–1.32)	1.06(1.01–1.11)	1.35(1.24–1.48)
Targeted vs. chemotherapy	7(78%)	4(80%)	0(0%)	0(0%)	1.16(1.06–1.28)	1.73(1.59–1.89)	1.20(1.08–1.33)	1.93(1.67–2.24)
Targeted vs. supportive care/placebo	6(60%)	9(90%)	0(0%)	2(20%)	1.07(1.02–1.13)	1.41(1.26–1.57)	1.07(1.02–1.12)	1.53(1.31–1.79)
Targeted-chemotherapy vs. chemotherapy	10(91%)	4(100%)	1(9%)	0(0%)	1.14(1.05–1.24)	1.1(1.07–1.13)	1.2(1.09–1.33)	1.16(1.06–1.27)
Targeted vs. targeted	13(81%)	14(100%)	0(0%)	3(21%)	1.05(1.03–1.06)	1.29(1.19–1.39)	1.08(1.05–1.11)	1.49(1.31–1.70)
Cancer type								
Breast cancer	13(72%)	18(95%)	2(11%)	2(11%)	1.07(1.03–1.10)	1.25(1.15–1.36)	1.09(1.05–1.14)	1.34(1.21–1.48)
Kidney cancer	10(83%)	8(100%)	0(0%)	1(13%)	1.07(1.04–1.10)	1.28(1.14–1.43)	1.09(1.06–1.13)	1.40(1.25–1.57)
Lung cancer	24(96%)	20(100%)	1(4%)	6(30%)	1.10(1.07–1.13)	1.34(1.27–1.41)	1.18(1.13–1.23)	1.57(1.42–1.73)
Melanoma	7(70%)	14(93%)	0(0%)	3(20%)	1.11(1.07–1.16)	1.24(1.16–1.32)	1.16(1.11–1.22)	1.39(1.28–1.51)
Multiple myeloma	6(100%)	7(88%)	3(50%)	3(38%)	1.03(1.02–1.05)	1.14(1.03–1.26)	1.15(1.03–1.23)	1.30(1.18–1.42)
Metastatic disease								
No	28(78%)	26(96%)	3(8%)	9(33%)	1.03(1.02–1.04)	1.17(1.11–1.22)	1.05(1.04–1.07)	1.30(1.21–1.39)
Yes	95(83%)	85(91%)	4(4%)	15(16%)	1.11(1.09–1.12)	1.31(1.27–1.36)	1.16(1.14–1.18)	1.47(1.41–1.53)
Updated time duration								
Short (< 12 months)	35(85%)	38(93%)	1(2%)	6(15%)	1.11(1.08–1.13)	1.29(1.23–1.36)	1.16(1.12–1.20)	1.43(1.34–1.52)
Medium (12–24 months)	36(77%)	26(87%)	0(0%)	3(10%)	1.10(1.07–1.12)	1.36(1.27–1.45)	1.12(1.10–1.15)	1.45(1.34–1.56)
Long (>24 months)	52(84%)	47(96%)	6(10%)	15(31%)	1.06(1.05–1.08)	1.22(1.18–1.27)	1.11(1.09–1.13)	1.41(1.32–1.50)
Information fraction of initial OS, or PFS/EFS								
Immature (< 50%)	43(74%)	10(91%)	5(9%)	3(27%)	1.04(1.03–1.05)	1.19(1.11–1.27)	1.07(1.05–1.09)	1.31(1.17–1.47)
Moderate (50%−75%)	49(86%)	42(89%)	0(0%)	15(32%)	1.11(1.09–1.13)	1.31(1.25–1.37)	1.15(1.12–1.18)	1.49(1.40–1.59)
Mature (>75%)	31(89%)	59(95%)	2(6%)	6(10%)	1.14(1.11–1.17)	1.27(1.22–1.32)	1.20(1.16–1.24)	1.39(1.33–1.46)

1. OS, overall survival; PFS/EFS, progression-free/event-free survival; 2. Only key subgroups are presented here; full subgroup information and heterogeneity details are provided in Supplementary Tables S4, S5.

Pooled RMST ratio was 1.08 (95% CI, 1.07–1.10) in initial analyses and 1.13 (1.11–1.14) in updated analyses. 82 of 150 comparisons initially showed a significant survival advantage for experimental groups, increasing to 93 after updates. Among 150 comparisons, 20 that were initially non-significant became significant after updating, while 9 lost prior significance. Detailed subgroup results and heterogeneity measures are provided in Table 1 and Supplementary Table S4.

**Figure 2:**
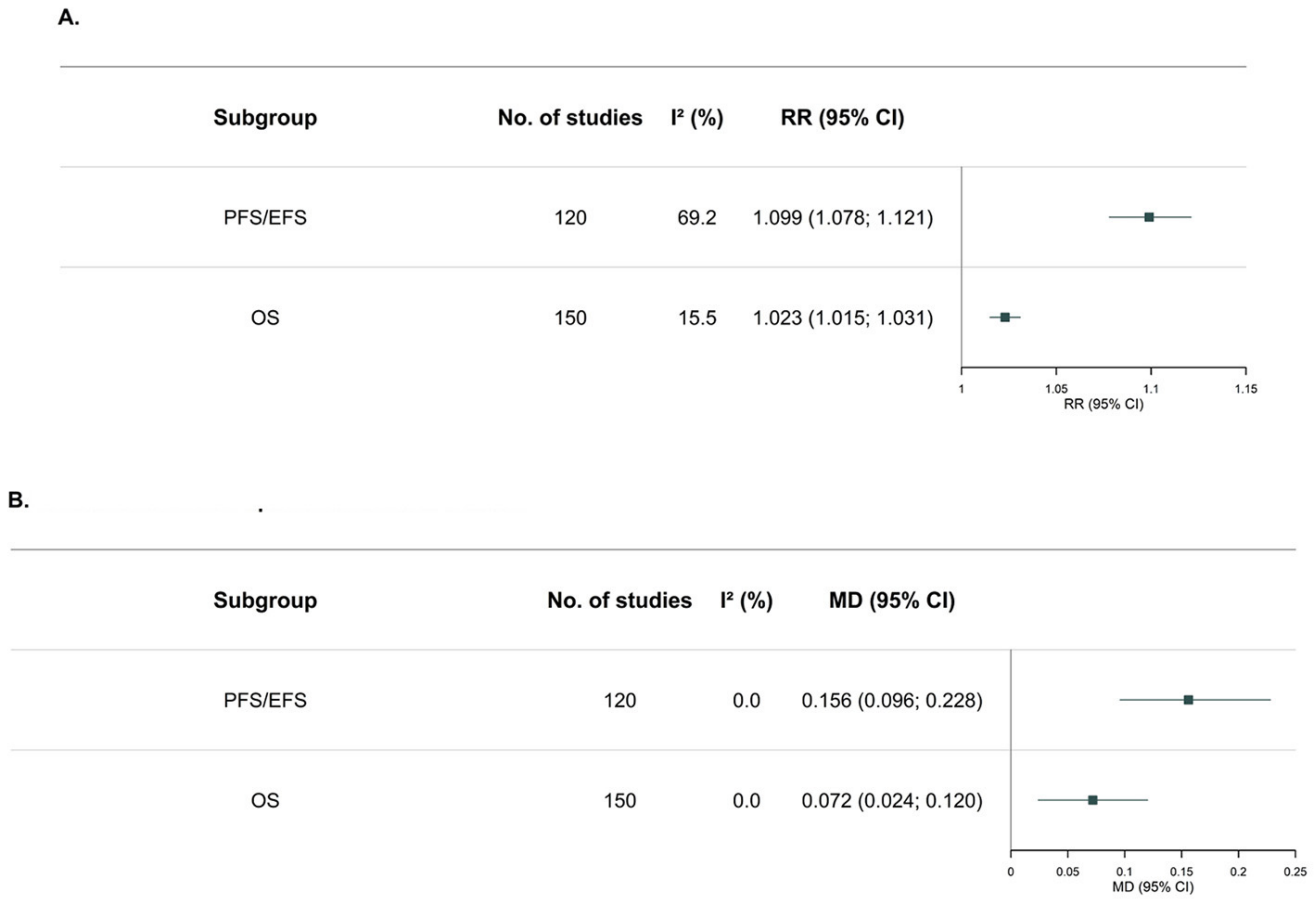
Changes in relative efficacy of FDA-approved treatments compared with controls between initial and updated analyses. nRMSD, difference between updated and initial normalized restricted mean survival differences; RR, ratio of the updated to initial restricted mean survival time (RMST) ratio; OS, overall survival; PFS/EFS, progression-free/event-free survival. An RR > 1 indicates that the relative efficacy of the experimental group compared with the control group increased after data updates, suggesting an apparent enhancement of survival benefit with more mature data. **(A)** Ratio of updated to initial RMST ratio. **(B)** Difference between updated and initial nRMSD.

**Figure 3:**
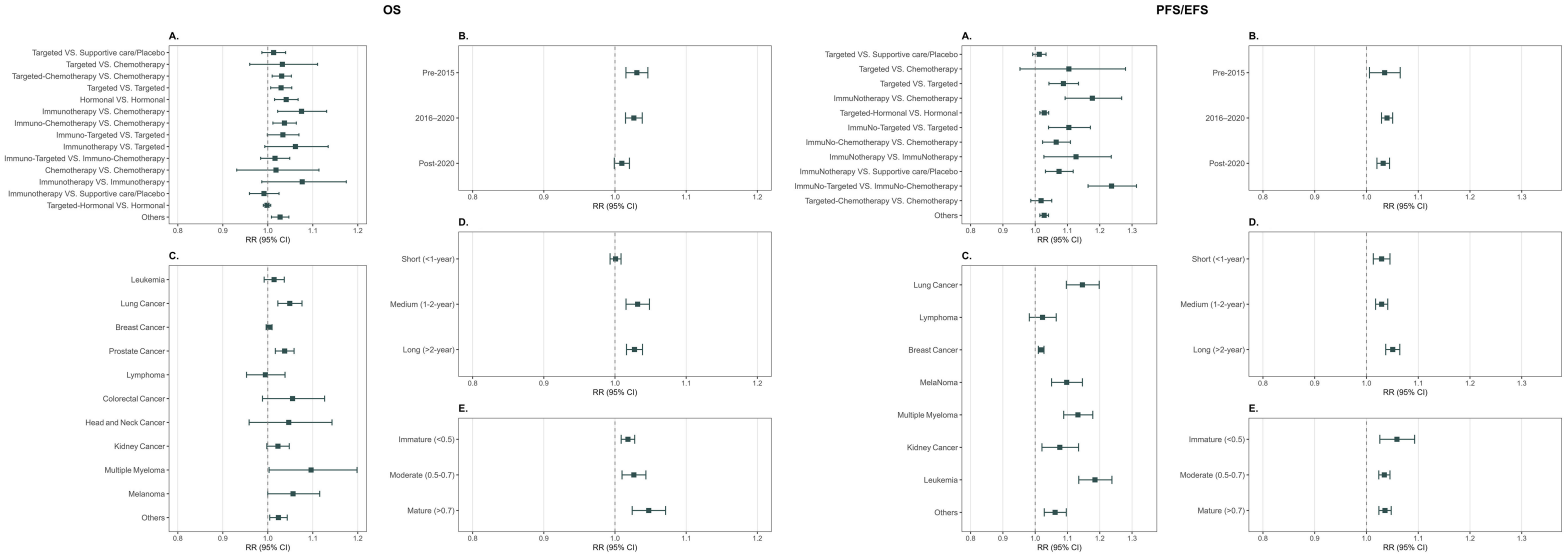
Subgroup analyses of changes in relative treatment effects between updated and initial analyses for OS and PFS/EFS. RR, ratio of updated to initial restricted mean survival time (RMST) ratio; 1. Only key subgroups are shown in this figure. Comprehensive subgroup results are provided in Supplementary Tables S10, S11; 2. An RR > 1 indicates that the relative efficacy of the experimental group compared with the control group increased after data updates, suggesting an apparent enhancement of survival benefit with more mature data. **(A)** Treatment group vs. control group. **(B)** Approve year. **(C)** Indication. **(D)** Update time duration. **(E)** Information fraction of the trial prior to the update.

As shown in Figure 2, updated analyses demonstrated an expanded relative OS advantage for FDA-approved innovative treatments over controls (unweighted mean RR, 1.052 [95% CI, 1.029–1.063]; meta-analytic RR, 1.023 [1.015–1.031], *I*^2^ = 15.5%), with increased relative efficacy observed in 127 of 150 comparisons (seven significantly increased) and decreased in 23 (one significantly decreased). Subgroup analyses supported this overall trend (Figure 3 and Supplementary Table S8), showing consistent improvements across treatment types, approval periods, and disease indications. Notably, greater OS gains were seen in trials of immunotherapy-based regimens and those with longer follow-up durations (>1 year). Trials with higher initial IF also exhibited more pronounced improvements. Across cancer types, the largest gains were observed in lung, breast, and colorectal cancers, while other malignancies showed similar but less marked trends. No substantial heterogeneity was detected among subgroups.

### Changes in PFS/EFS between initial and updated results

A total of 120 paired comparisons were included. PH assumption was satisfied in 58 comparisons and violated in 62. Median maximum follow-up increased from 25.8 months (IQR, 17.8–34.3) in initial reports to 49.7 months (33.9–67.4) in updated analyses. Median censoring rates decreased from 0.38 (0.19–0.63) to 0.25 (0.11–0.47) in experimental groups, and from 0.20 (0.06–0.40) to 0.11 (0.04–0.26) in control groups. Further details on study characteristics and subgroup distributions are provided in Table 1.

Pooled RMST ratio between FDA-approved innovative treatments and controls for PFS was 1.28 (95% CI, 1.24–1.31) in initial analyses and 1.42 (1.37–1.48) in updated analyses. Initially, 106 of 120 (88.3%) comparisons showed a significant advantage for experimental group over control, which rose to 113 (94.2%) after data updates. Nine comparisons that were initially non-significant became significant in favor of the experimental arm, while two lost prior significance. Further subgroup and heterogeneity information is presented in Table 1 and Supplementary Table S5.

Updated evidence showed a significant improvement in RMST ratios for PFS/EFS [unweighted mean RR, 1.131 (95% CI, 1.102–1.153); meta-analytic RR, 1.099 (95% CI, 1.078–1.121), *I*^2^ = 69.2%]. RMST ratio increased in 111 of 120 comparisons (24 significant) and decreased in 9 (none were significant). Subgroup analyses confirmed this consistent trend, with no reversal of benefit (Figure 3 and Supplementary Table S9). Greater improvements were observed in trials evaluating immunotherapy-based and targeted regimens and in those with longer follow-up durations. Among cancer types, lung, breast, and kidney cancers showed the most pronounced gains, while hematologic malignancies exhibited smaller effects.

### Meta-regression results

**Figure 4:**
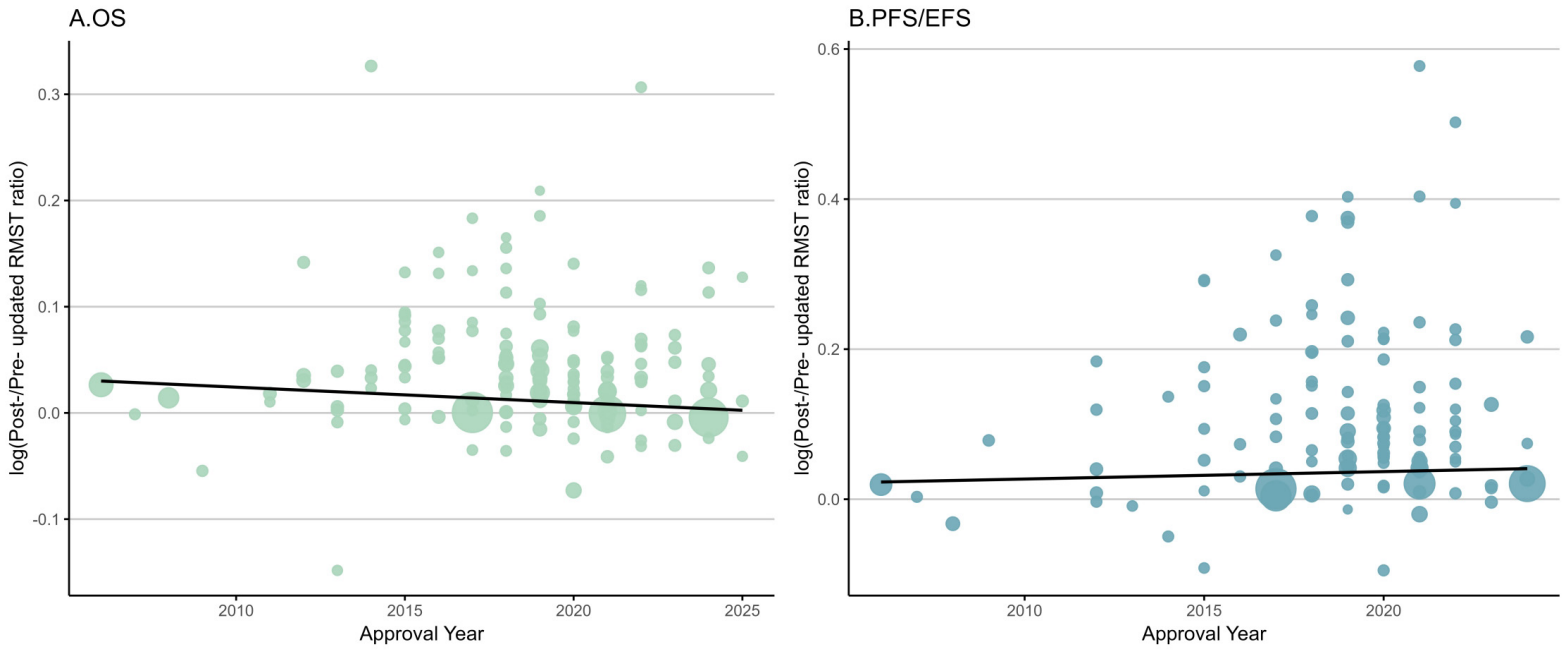
Meta-regression of changes in relative treatment effects between updated and initial analyses by year of FDA approval. AE, adverse event; RR, ratio of the updated to initial restricted mean survival time (RMST) ratio; OS, overall survival; PFS/EFS, progression-free/event-free survival. Regression equations: **(A)** OS, log(RR) = 2.94 – 0.002 × year; **(B)** PFS/EFS, log(RR) = −1.961 + 0.001 × year.

As shown in Figure 4, the univariable meta-regression by FDA approval year revealed a modest but statistically significant decline in the RR for OS over time [coefficient [β] = −0.002, *p* = 0.01], indicating that the relative improvement in OS after data updates was slightly smaller for more recently approved agents. No significant temporal association was observed for PFS/EFS (β = 0.001, *p* = 0.30). Details are in Supplementary Tables S10, S11.

Multivariable meta-regression findings are summarized in Supplementary Tables S12, S13, with detailed results presented in Supplementary Result S1. Briefly, for efficacy outcomes, trials in which the control arm's initial data were less mature or its updated data became more mature showed greater apparent relative survival benefits for experimental groups after data updates. Similarly, for PFS/EFS, shorter initial follow-up was significantly associated with a larger increase in relative efficacy at updated analyses.

For endpoints with potential heterogeneity, meta-regression effectively reduced between-study variability. Specifically, for PFS/EFS, heterogeneity decreased markedly (*I*^2^ from 69.2% to 21.7%), accounting for approximately 74% of the total variability across studies.

### Sensitivity analysis results

Results of sensitivity analyses were consistent with the primary analyses. As shown in Figure 2, across 120 comparisons reporting PFS/EFS, updated analyses demonstrated an increase of 0.156 months per year (95% CI, 0.096–0.228; *I*^2^ = 0%) in nRMSD between experimental and control groups, indicating an enhanced relative survival benefit compared with initial analyses. For OS, the corresponding increase in nRMSD was 0.072 months per year (0.024–0.120; *I*^2^ = 0%) in updated analyses.

When RMSTs were truncated at 95% and 90% of the origin truncation time, meta-analytic RRs for OS remained stable at 1.023 (95% CI, 1.015–1.030) and 1.022 (1.015–1.030), respectively. For PFS/EFS, the corresponding RRs were 1.10 (95% CI, 1.078–1.121) and 1.10 (1.079–1.121).

## Discussion

In this systematic retrospective analysis, the relative efficacy of FDA-approved innovative treatments compared with controls improved consistently after data updates. Mean RR was 1.052 (95% CI, 1.029–1.063) for OS and 1.131 (1.102–1.153) for PFS/EFS, indicating average increases of 5.2% and 13.1% in RMST ratios between innovative therapies and controls. Meta-analytic RRs [1.023 (1.015–1.031) for OS and 1.099 (1.078–1.121) for PFS/EFS] were also statistically significant. These improvements were consistent across treatment types and indications, with greater gains in immunotherapy-based and targeted regimens, especially in lung, breast, and colorectal cancers. Sensitivity analyses demonstrated increases in relative survival time between the experimental and control groups of 0.072 months/year for OS and 0.156 months/year for PFS/EFS. These observations should be interpreted within the analyzed sample, as trials without updated outcomes or reconstructable curves could not be evaluated and may bias estimates toward favorable durability.

For innovative therapies, trials with less mature control-arm data at baseline or with longer updated follow-up showed greater relative survival gains. In our analysis, 26.7% of OS comparisons and 51.7% of PFS/EFS comparisons violated the PH assumption in either the initial or updated reports. Therefore, we adopted RMST to avoid the potential misinterpretation associated with the HR under non-PH conditions (19). In both OS and PFS/EFS analyses, updated reports demonstrated a greater statistically significant survival advantage for the experimental groups. To address potential bias in RMST estimation due to sparse tail data, sensitivity analyses with follow-up truncated at 95% and 90% of τ further supported the robustness of the primary findings.

Overall heterogeneity in our study was low. Although moderate heterogeneity was observed in PFS/EFS analyses, most of it was accounted for by meta-regression. Heterogeneity observed for PFS/EFS primarily reflected variation in effect magnitude rather than direction (only 1.7% of comparisons showed opposite trends). Heterogeneity may have been influenced by the duration of data updates. When relative differences were standardized per unit time using nRMSD, heterogeneity for both OS and PFS decreased to zero. Collectively, the low heterogeneity underscores the robustness and generalizability of our findings across cancer types and trial designs (25).

A potential concern is whether increasing RMST ratios over longer follow-up reflect true clinical benefit rather than artifacts of extended observation, evolving censoring, or changing τ. Several findings argue against such artifacts. The RMST ratio is dimensionless and not mechanically inflated by τ; for RR to increase, the experimental curve must genuinely diverge further from the control beyond the initial horizon. Re-truncation at 95% and 90% of τ yielded virtually identical RRs, and per-year-normalized nRMSD reduced heterogeneity to *I*^2^ = 0%, indicating that gains scaled with follow-up rather than tail instability. Moreover, declining censoring rates reflect increased event maturity rather than bias, and meta-regression showed that larger RRs were linked to less mature control-arm data at baseline—a pattern consistent with delayed clinical separation rather than a generic “longer-is-larger” effect.

The stronger relative efficacy observed after data updates likely reflects the interplay between treatment innovation and control-arm limitations. Blumenthal et al. (26) analyzed 25 trials and found that survival curves for immunotherapy separated only after several months, suggesting that efficacy became more pronounced as the data matured. Chen et al. (27) provided methodological evidence that immunotherapies exhibit delayed clinical effects and late curve separation. Xu et al. (28) confirmed the long-term advantages of multi-mechanism regimens, while Das et al. (29) demonstrated that novel molecular targets can delay resistance and prolong survival. Tan et al. (30) analyzed 32 combination trials and reported consistent improvement in both PFS and OS with multi-mechanism regimens compared with monotherapy or chemotherapy. Pocock et al. (31) showed that premature analyses may underestimate true treatment effects due to incomplete event capture, and Hellmann et al. (32) emphasized that median survival underestimates the true benefit of therapies inducing durable responses, reflecting the “long-tail” efficacy sustained over time. Collectively, these findings support the interpretation that innovative therapies often produce delayed but durable benefits, whereas control regimens may experience early resistance, leading to diminished long-term efficacy.

The observed increase in RMST ratios should be interpreted in light of several interrelated biases. Survivorship bias is intrinsic to our design: by anchoring inclusion on successful FDA approvals, our cohort is enriched for therapies that had already demonstrated promising initial efficacy, while trials terminated early for futility or failing to gain approval are not represented; their inclusion would likely attenuate the observed gains. Selective availability of updated analyses is a substantial concern, as many eligible RCTs lacked publicly available updates. Sponsors have stronger incentives to disseminate updates that confirm or amplify benefit, whereas trials with attenuated effects are more likely to experience delayed publication or non-publication. This asymmetry is most pronounced for endpoints requiring the longest follow-up. Publication and reporting bias may further compound these effects. Together, these biases share a common direction, each tending to overestimate the apparent durability of treatment benefit, and the magnitude of efficacy evolution reported here should therefore be regarded as a plausible upper bound. Notably, our meta-regression showed that trials with less mature control-arm data at baseline exhibited larger apparent RR, suggesting that part of the observed evolution may also reflect control-arm maturation rather than experimental-arm gain alone. Nevertheless, the directional consistency across subgroups (treatment classes, cancer types, approval eras, PH/non-PH strata), the robustness across sensitivity analyses, and the modest pooled OS RR (1.023) collectively suggest a genuine underlying signal rather than artifact. Future research incorporating unpublished updates and regulatory-held datasets is needed for a less biased estimate.

## Public health significance

Beyond their methodological and regulatory implications, our findings carry substantial public health relevance. Oncology drugs approved by the FDA are rapidly diffused into clinical practice, influence international regulatory decisions, shape clinical guidelines, and determine insurance coverage and reimbursement at the population level. Because many approvals rely on immature evidence, uncertainty at the regulatory stage propagates downstream to healthcare systems, payers, clinicians, and ultimately patient populations, with direct consequences for healthcare expenditure, equitable access, and population-level cancer outcomes. By systematically demonstrating that the relative benefit of FDA-approved oncology therapies generally strengthens rather than diminishes with evidence maturation, our results provide population-level reassurance that expedited approval pathways, on average, deliver durable clinical value. At the same time, the heterogeneity observed across trials—and the small subset of comparisons in which benefit attenuated—highlights the public health imperative of robust post-marketing surveillance, transparent evidence updating, and continuous reassessment of benefit-risk balance after approval.

## Implications

The FDA framework already includes dynamic elements such as Accelerated Approval, Post-Marketing Requirements, and Project Confirm. Our findings support further systematizing these mechanisms rather than restructuring them. From a public health perspective, regulatory practice should pay attention to an evidence-maturity-based dynamic management model that integrates periodic reassessment, and strengthened post-marketing surveillance of long-term efficacy. For medicines initially approved on surrogate or intermediate endpoints, predefined timelines for evidence updates and outcome verification are essential. Continuous incorporation of mature clinical and real-world data can ensure that regulatory decisions reflect the true balance between benefit and risk (33, 34).

Analytical approaches should also evolve. Because many modern oncology trials exhibit non-PH, reliance solely on HRs may obscure long-term efficacy. Complementary time-integrated measures such as RMST offer a clearer depiction of treatment benefit as it evolves over time (35).

Pricing and reimbursement mechanisms should align with long-term population-level value. Adaptive or outcome-based payment models can link cost to confirmed clinical benefit, allowing drug prices to evolve with evidence maturity and supporting equitable access to effective oncology therapies across diverse healthcare settings. Enhancing transparency and international collaboration—including data sharing and coordinated reassessment across regulatory agencies—can further support consistent, life-cycle governance of oncology drugs.

Clinicians should recognize the time-dependent nature of therapeutic effects and maintain extended follow-up to capture delayed but durable survival benefits. From a research perspective, longer follow-up and greater analytical robustness are needed to detect delayed efficacy and late divergences of survival curves. Continuous evidence renewal, integrating mature trial findings and real-world outcomes, is essential to ensure that rapid access to innovative therapies is matched by durable benefit and sustained clinical value at both the individual and population levels.

## Limitation

First, our analysis was subject to selection bias on two levels: only RCTs with published updates and reconstructable Kaplan-Meier curves were included. Trials with sustained or favorable benefit are more likely to release updates and to be reported in sufficient detail for IPD reconstruction, likely biasing pooled estimates toward greater durability of benefit. Our findings should therefore be regarded as descriptive of trials with continued reporting rather than a comprehensive validation of FDA approvals. Second, reconstructed IPD were derived from Kaplan-Meier curves; while validation confirmed high concordance, minor estimation errors cannot be fully excluded. Third, although multiple subgroup and sensitivity analyses supported the robustness of RMST-based longitudinal comparisons, residual influence of follow-up duration on RMST estimation cannot be entirely excluded. Finally, our analysis focused solely on FDA-approved drugs, and future research should incorporate approvals from other regulatory agencies to enhance global applicability.

## Conclusion

This study provides the first comprehensive evaluation of how efficacy outcomes evolve from initial to updated datasets supporting FDA oncology drug approvals. Updated evidence consistently showed greater survival benefits. These findings indicate that most approved oncology drugs with updated data achieve more durable benefits over time, though selection bias inherent to the included trials precludes a global validation of FDA approval decisions. However, the evolving nature of these treatment effects highlights the ongoing need for dynamic oversight of long-term clinical value. Our findings support building upon the dynamic elements of the existing FDA framework through standardized re-evaluation timelines and integration of mature efficacy data.

## Data Availability

The original contributions presented in the study are included in the article/Supplementary material, further inquiries can be directed to the corresponding author.
