# Supplementary material for: Evolution of clinical benefit in cancer trials: comparing pivotal approval data with updated efficacy
Source: Front Public Health. 2026 Jul 3;14:1876451. doi: 10.3389/fpubh.2026.1876451 (PMC13375909; doi:10.3389/fpubh.2026.1876451)
Supplement: Supplementary file 1 [file Data_Sheet_1.docx]

**Supplementary Material**

[Figure S1 Study flow chart 2](#_Toc27912)

[Table S1. Prespecified variables in meta-regression analyses across OS, PFS (including EFS) 3](#_Toc13555)

[Table S2 Prespecified subgroups and rationale for stratification across OS, PFS (including EFS), and AE analyses 5](#_Toc21948)

[Table S3 Characteristics of 165 clinical trials included in the analysis 7](#_Toc3379)

[Table S4 Overall and Subgroup Meta-analysis of RMST ratio for OS before and after data updates 22](#_Toc20234)

[Table S5 Overall and Subgroup Meta-analysis of RMST ratio for PFS/EFS before and after data updates 25](#_Toc28611)

[Table S6 The distribution of trial-specific τ values for OS 28](#_Toc15884)

[Table S7 The distribution of trial-specific τ values for EFS/PFS 31](#_Toc22599)

[Table S8 Overall and Subgroup Meta-analysis of RR (Ratio of updated RMST ratio to initial RMST ratio) for OS 35](#_Toc30465)

[Table S9 Overall and Subgroup Meta-analysis of RR (Ratio of updated RMST ratio to initial RMST ratio) for PFS/EFS 38](#_Toc7301)

[Table S10 Univariable meta-regression results of overall survival (OS) by individual covariates 41](#_Toc2321)

[Table S11 Univariable meta-regression results of PFS/EFS by individual covariates 42](#_Toc17813)

[Table S12 Multivariable meta-regression results of OS 43](#_Toc32061)

[Table S13 Multivariable meta-regression results of PFS/EFS 44](#_Toc29594)

[Result S1 Detailed results for multivariable meta-regression analyses 45](#_Toc18875)

[Reference 46](#_Toc10269)

# Figure S1 Study flow chart

Trials used for FDA drug approvals from January 1, 2006, to September 1, 2025 (n =524)

Excluded: n = 33

- Records of non-oncology, supportive care, and diagnostic agents

Titles and abstracts screening

(n =491)

Excluded: n =156

- Trials that did not have published updated curves (OS or PFS or DFS or RFS or EFS)

Full-text articles screening

(n =329)

Excluded: n = 162

- Non-randomized uncontrolled trials

- Trials not in phase Ⅱ/Ⅲ

Trials finally included in our study

(n =165)

**Identification**

**Screening**

**Eligibility**

**Inclusion**

Excluded: n =8

- Trials that have only reported survival curves (OS, PFS, DFS, RFS, or EFS) without providing the number at risk

# Table S1. Prespecified variables in meta-regression analyses across OS, PFS (including EFS)

| Endpoint | Variable | Variable type | Definition / Coding | Rationale or interpretation |
| --- | --- | --- | --- | --- |
| OS / PFS | Proportional‑hazards status | Categorical (binary) | 0 = assumption met; 1 = violated in either pre‑ or post‑analysis | Checks whether violation of the PH assumption influences consistency of survival outcomes |
| OS / PFS | FDA approval year | Continuous (numeric) | Calendar year of FDA approval for the investigational regimen | Represents therapeutic‑era or time‑trend effect |
| OS / PFS | Five‑year disease survival rate | Continuous (proportion 0–1) | Background long‑term survival rate of the underlying cancer type | The 5-year survival rates by indication were obtained from the SEER database (https://seer.cancer.gov/) |
| OS / PFS | Trial phase | Categorical (Phase II vs Phase III) | Phase II = early‑stage exploratory; Phase III = confirmatory | Proxy for trial rigor and maturity of evidence |
| OS / PFS | Treatment line | Categorical (binary) | First‑line = 0; Later‑line = 1 (subsequent or relapsed setting) | Captures previous therapy exposure and clinical context |
| OS / PFS | Metastatic setting | Categorical (binary) | Non‑metastatic = 0; Metastatic = 1 | Represents baseline disease extent and prognosis |
| OS / PFS | Blinding status | Categorical (binary) | Open‑label = 0; Blinded = 1 | Indicator of potential performance / assessment bias |
| OS | Crossover allowance | Categorical (binary) | No = 0; Yes = 1 | Tests whether crossover diluted differences |
| OS / PFS | Information fraction | Continuous (ratio 0–1) | Observed / planned event proportion | Quantifies maturity of efficacy data^1^ |
| OS / PFS | Follow‑up duration | Continuous (months) | Difference between post‑ and pre‑cutoff times | Reflects observation time and data maturity |
| OS / PFS | Data cutoff time | Continuous (months) | Cutoff times used in RMST calculation, including both the initial and updated analyses | Indicator of time in calculating RMST |
| OS / PFS | Censoring rates | Continuous (proportion 0–1) | Pre‑ and post‑analysis censoring rates for treatment / control arms | Quantifies right‑censoring of survival data |
| OS / PFS | Trial sample size | Categorical (ordinal) | Small (< 100)=1, Medium (100–600)=2, Large (> 600)=3 | Represents study size and statistical power of individual trials |

Note, OS = overall survival; PFS = progression-free survival (including EFS)

# Table S2 Prespecified subgroups and rationale for stratification across OS, PFS (including EFS), and AE analyses

| Endpoint | Subgroup variable | Levels / Coding rule | Rationale / Reference |
| --- | --- | --- | --- |
| OS / PFS | PH status | 0 = PH met in both initial and updated analyses; 1 = violated in either | To evaluate whether violation of the proportional hazards assumption affects consistency of survival outcome comparisons |
| OS / PFS | FDA approval year | ≤ 2015 (Pre‑Immunotherapy era); 2016–2020 (Immune‑oncology expansion); ≥ 2021 (Modern combination and biomarker‑driven era) | Reflects chronological evolution of oncology therapeutics and regulatory landscapes |
| OS / PFS | 5‑year survival rate | Low (< 30%), Intermediate (30–69%), High (≥ 70%) | The 5-year survival rates by indication were obtained from the SEER database (https://seer.cancer.gov/), with classification criteria based on Siegel et al^2^ |
| OS / PFS | Trial sample size | Small (< 100), Medium (100–600), Large (> 600) | Represents trial scale, event maturity, and statistical precision^3^ |
| OS / PFS | Treatment line | First‑line vs later‑line | Reflects line of therapy and prior treatment exposure |
| OS / PFS | Treatment category | Defined by mechanism of experimental and control regimens (e.g., IO vs chemotherapy vs targeted therapy); regimens with < 4 trials pooled as “Others” | Captures mechanistic class and control type differences, not specific drug names |
| OS / PFS | Trial phase | Phase II vs Phase III | Distinguishes trial design rigor and maturity of clinical evidence |
| OS / PFS | Blinding | Blinded vs Open‑label | Evaluates risk of performance and detection bias |
| OS/ SAE | Crossover allowance | Yes vs No | Tests whether patient crossover attenuates between‑group effects |
| OS / PFS | Disease type | Rare (< 5 studies) merged as “Others” | Reduces heterogeneity from small tumor groups |
| OS / PFS | Metastatic status | Metastatic vs Non‑metastatic | Accounts for disease stage and baseline prognosis |
| OS / PFS | Follow‑up duration | Short (< 12 mo), Medium (12–24 mo), Long (> 24 mo) | Reflects timing and completeness of endpoint assessment |
| OS / PFS | Information fraction (IF) | < 50% (Immature), 50–75% (Moderate), > 75% (Mature) | Indicates data maturity by observed / planned event proportion^1^ |

Note, OS = overall survival; PFS = progression-free survival (including EFS)

# Table S3 Characteristics of 165 clinical trials included in the analysis

| No of trial | Trial | NCT | Blinding, Phase | FDA Approve Year | Indication | Treatment Regimen | Control Regimen | Treatment Group Sample Size | Control Regimen Sample Size |
| --- | --- | --- | --- | --- | --- | --- | --- | --- | --- |
| 1 | ACOSOG Z9001 | NCT00041197 | double-blind, phase 3 | 2008 | primary gastrointestinal stromal tumor | Imatinib mesylate | Placebo | 359 | 354 |
| 2 | ADAURA | NCT02511106 | double-blind, phase 3 | 12/18/2020 | EGFR-mutated (Ex19del or L858R) non–small-cell lung cancer | Osimertinib | Placebo | 339 | 343 |
| 3 | ADMIRAL | NCT02421939 | open-label, phase 3 | 11/28/2018 | FLT3-mutated acute myeloid leukemia | Gilteritinib | salvage chemotherapy | 247 | 124 |
| 4 | AETHERA | NCT01100502 | double-blind, phase 3 | 11/10/2022 | Classical Hodgkin lymphoma that is relapsed or primary refractory | Brentuximab vedotin | Placebo | 165 | 164 |
| 5 | AG120-C-005 | NCT02989857 | double-blind, phase 3 | 8/25/2021 | IDH1‑mutant cholangiocarcinoma | Ivosidenib | placebo | 124 | 61 |
| 6 | AG120-C-009 | NCT03173248 | double-blind, phase 3 | 5/25/2022 | Newly diagnosed IDH1-mutated acute myeloid leukemia | Ivosidenib+azacitidine | Azacitidine | 72 | 74 |
| 7 | ALCANZA | NCT01578499 | open-label, phase 3 | 11/16/2018 | CD30-positive cutaneous T-cell lymphoma | Brentuximab vedotin | methotrexate | 64 | 64 |
| 8 | ALEX | NCT02075840 | open-label, phase 3 | 11/6/2017 | ALK-positive non–small-cell lung cancer | Alectinib | Crizotinib | 152 | 151 |
| 9 | ALFA-0701 | NCT00927498 | open-label, phase 3 | 2017 | acute myeloid leukemia | chemotherapy+fractionated-dose gemtuzumab ozogamicin | chemotherapy | 139 | 139 |
| 10 | ALTA-1L | NCT02737501 | open-label, phase 3 | 5/22/2020 | ALK-positive non–small-cell lung cancer | Brigatinib | Crizotinib | 137 | 138 |
| 11 | APHINITY | NCT01358877 | double-blind, phase 3 | 12/20/2017 | early HER2-positive breast cancer | Pertuzumab+trastuzumab+chemotherapy | trastuzumab+chemotherapy | 2400 | 2405 |
| 12 | ARAMIS | NCT02200614 | double-blind, phase 3 | 7/30/2019 | castration-resistant prostate cancer | Darolutamide+androgen-deprivation therapy | androgen-deprivation therapy | 955 | 554 |
| 13 | ARCHES | NCT02677896 | double-blind, phase 3 | 12/16/2019 | Metastatic hormone-sensitive prostate cancer | Enzalutamide+androgen deprivation therapy | androgen deprivation therapy | 574 | 576 |
| 14 | ASCENT | NCT02574455 | open-label, phase 3 | 4/7/2021 | Relapsed or refractory metastatic triple-negative breast cancer | Sacituzumab govitecan | Single-agent chemotherapy of physician's choice | 235 | 233 |
| 15 | ATTRACTION-3 | NCT02569242 | open-label, phase 3 | 5/27/2022 | Unresectable advanced or recurrent esophageal squamous cell carcinoma | Nivolumab | Investigator's choice single-agent chemotherapy | 210 | 209 |
| 16 | AUGMENT | NCT01938001 | double-blind, phase 3 | 5/28/2019 | indolent non-Hodgkin lymphoma | Lenalidomide+rituximab | rituximab | 178 | 180 |
| 17 | BEACONCRC (DOU-CON) | NCT02928224 | open-label, phase 3 | 4/8/2020 | Metastatic colorectal cancer with BRAF V600E mutation | encorafenib + binimetinib + cetuximab | cetuximab + irinotecan or cetuximab + FOLFIRI | 224 | 221 |
| 17 | BEACONCRC (TRI-CON) | NCT02928224 | open-label, phase 3 | 4/8/2020 | Metastatic colorectal cancer with BRAF V600E mutation | encorafenib + cetuximab | cetuximab + irinotecan or cetuximab + FOLFIRI | 220 | 221 |
| 18 | BOLERO-2 | NCT00863655 | double-blind, phase 3 | 7/24/2012 | hormone receptor–positive, HER2‑negative advanced/metastatic breast cancer | Everolimus plus exemestane | Exemestane | 485 | 239 |
| 19 | Bonner | NCT00004227 | open-label, phase 3 | 4/6/2021 | Locoregionally advanced (stage III–IV), non-metastatic, measurable squamous-cell carcinoma | Cetuximab | Radiotherapy | 211 | 213 |
| 20 | BREAK-3 | NCT01227889 | open-label, phase 3 | 6/22/2022 | unresectable stage III or stage IV BRAF V600E–mutated metastatic melanoma | Dabrafenib | Dacarbazine | 187 | 63 |
| 21 | BREAKWATER | NCT04607421 | open-label, phase 3 | 12/20/2024 | metastatic colorectal cancer with BRAF V600E mutation (stage IV) | Encorafenib+mFOLFOX6 | Standard of Care | 236 | 243 |
| 22 | BRIGHT AML 1003 | NCT01546038 | open-label，phase 2 | 2018 | Newly diagnosed acute myeloid leukemia | glasdegib plus low-dose cytarabine | low-dose cytarabine | 88 | 44 |
| 23 | CABOSUN | NCT01835158 | open-label, phase 2 | 12/19/2017 | Metastatic clear cell renal cell carcinoma | Cabozantinib | Sunitinib | 79 | 78 |
| 24 | CANDOR | NCT03158688 | open-label, phase 3 | 8/20/2020 | Relapsed or refractory multiple myeloma | Carfilzomib+dexamethasone+daratumumab | Carfilzomib + dexamethasone | 312 | 154 |
| 25 | CASPIAN | NCT03043872 | open-label, phase 3 | 3/27/2020 | Extensive-stage small-cell lung cancer | Durvalumab+platinum–etoposide | Platinum–etoposide | 268 | 269 |
| 26 | CASSIOPEIA | NCT02541383 | open-label, phase 3 | 9/26/2019 | diagnosed multiple myeloma | Daratumumab + bortezomib + thalidomide + dexamethasone | Bortezomib + thalidomide + dexamethasone | 543 | 542 |
| 27 | CASTOR | NCT02136134 | open-label, phase 3 | 7/30/2024 | refractory multiple myeloma | Daratumumab+bortezomib+dexamethasone | Bortezomib+dexamethasone | 251 | 247 |
| 28 | CheckMate 816 | NCT02998528 | open-label, phase 3 | 3/4/2022 | Resectable Non-Small Cell Lung Cancer | Nivolumab+platinum-doublet chemotherapy | Platinum-doublet chemotherapy | 179 | 179 |
| 29 | CheckMate 9LA | NCT03215706 | open-label, phase 3 | 5/26/2020 | advanced (stage IV or recurrent) non–small cell lung cancer | Nivolumab+ipilimumab+chemotherapy | chemotherapy | 361 | 358 |
| 30 | CheckMate025 | NCT01668784 | open-label, phase 3 | 2015 | Advanced or metastatic clear-cell renal-cell carcinoma | Nivolumab | Everolimus | 410 | 411 |
| 31 | CheckMate057 | NCT01673867 | open-label, phase 3 | 2015 | Advanced or recurrent nonsquamous non–small-cell lung cancer | Nivolumab | Docetaxel | 292 | 290 |
| 32 | CheckMate141 | NCT02105636 | open-label, phase 3 | 2016 | refractory squamous-cell carcinoma of the head and neck | Nivolumab | systemic therapy | 240 | 121 |
| 33 | CheckMate214 | NCT02231749 | open-label, phase 3 | 4/16/2018 | Previously untreated advanced (metastatic) clear-cell renal-cell carcinoma | Nivolumab | Sunitinib | 550 | 546 |
| 34 | CheckMate227 | NCT02477826 | open-label, phase 3 | 5/15/2020 | advanced non–small-cell lung cancer | Nivolumab+ipilimumab | Platinum‑doublet chemotherapy | 396 | 397 |
| 35 | CheckMate238 | NCT02388906 | double-blind, phase 3 | 12/20/2017 | stage IV melanoma | Nivolumab | Ipilimumab | 453 | 453 |
| 36 | CHECKMATE-274 | NCT02632409 | double-blind, phase 3 | 8/19/2021 | High‑risk muscle‑invasive urothelial carcinoma | Nivolumab | Placebo | 353 | 356 |
| 37 | CHECKMATE-648 (NC-C) | NCT03143153 | open-label, phase 3 | 5/31/2022 | metastatic esophageal squamous-cell carcinoma | Nivolumab+Chemotherapy | Chemotherapy | 321 | 324 |
| 37 | CHECKMATE-648 (NI-C) | NCT03143153 | open-label, phase 3 | 5/31/2022 | metastatic esophageal squamous-cell carcinoma | Nivolumab+Ipilimumab | Chemotherapy | 325 | 324 |
| 38 | CheckMate649 | NCT02872116 | open-label, phase 3 | 4/16/2021 | HER2‑negative gastric, gastro‑oesophageal junction, or oesophageal adenocarcinoma | Nivolumab+chemotherapy | Chemotherapy | 789 | 792 |
| 39 | CheckMate743 | NCT02899299 | open-label, phase 3 | 10/2/2020 | malignant pleural mesothelioma | Nivolumab+ipilimumab | Platinum+pemetrexed | 303 | 302 |
| 40 | CheckMate9ER | NCT03141177 | open-label, phase 3 | 1/22/2021 | advanced renal-cell carcinoma with a clear-cell component | Nivolumab+cabozantinib | Sunitinib | 323 | 328 |
| 41 | CLEAR | NCT02811861 | open-label, phase 3 | 8/10/2021 | advanced renal cell carcinoma with a clear-cell component | Lenvatinib 20 mg+Pembrolizumab 200 mg | Sunitinib | 355 | 357 |
| 42 | CLEOPATRA | NCT00567190 | double-blind, phase 3 | 6/8/2012 | HER2-positive locally recurrent, unresectable, or metastatic breast cancer | Pertuzumab + Trastuzumab + Docetaxel | Trastuzumab + Docetaxel | 402 | 406 |
| 43 | CLL | NCT02242942 | open-label, phase 3 | 5/15/2019 | untreated chronic lymphocytic leukemia | Venetoclax+obinutuzumab | Chlorambucil–Obinutuzumab | 216 | 216 |
| 44 | CLL8 | NCT00281918 | open-label, phase 3 | 1/23/2019 | CD20-positive chronic lymphocytic leukemia | Fludarabine + Cyclophosphamide + Rituximab | Fludarabine + Cyclophosphamide | 408 | 409 |
| 45 | CLTR0310-301 | NCT01696084 | open-label，phase phase 3 | 8/3/2017 | Newly diagnosed secondary acute myeloid leukemia | CPX-351 (cytarabine and daunorubicin) Liposome | CPX-351 and daunorubicin | 153 | 156 |
| 46 | coBRIM | NCT01689519 | double-blind，phase phase 3 | 2015 | melanoma | Vemurafenib + cobimetinib | Vemurafenib + placebo | 247 | 248 |
| 47 | CodeBreaK 300 (240-C) | NCT05198934 | open-label, phase 3 | 1/16/2025 | metastatic colorectal cancer with KRAS G12C mutation | 240-mg Sotorasib–Panitumumab | Standard Care | 53 | 54 |
| 47 | CodeBreaK 300 (960-C) | NCT05198934 | open-label, phase 3 | 1/16/2025 | metastatic colorectal cancer with KRAS G12C mutation | 960-mg Sotorasib–Panitumumab | Standard Care | 53 | 54 |
| 48 | COLUMBUS (ENB-EN) | NCT01909453 | open-label, phase 3 | 6/27/2018 | metastatic cutaneous melanoma with BRAFV600E or BRAFV600K mutation | Encorafenib 450 mg+binimetinib 45 mg | Encorafenib | 192 | 194 |
| 49 | COLUMBUS (ENB-VEM) | NCT01909453 | open-label, phase 3 | 6/27/2018 | metastatic cutaneous melanoma with BRAFV600E or BRAFV600K mutation | Encorafenib 450 mg+binimetinib 45 mg | Vemurafenib | 192 | 191 |
| 50 | COMBI-AD | NCT01682083 | double-blind，phase phase 3 | 4/30/2018 | completely resected, stage III melanoma with BRAF V600E or V600K mutations | Dabrafenib plus trametinib | Placebo | 438 | 432 |
| 51 | COMBI-d | NCT01584648 | double-blind, phase 3 | 6/22/2022 | Unresectable stage IIIC or stage IV BRAF V600E or V600K-mutant metastatic melanoma | Dabrafenib+Trametinib | Dabrafenib | 211 | 212 |
| 52 | COMPLEMENT 1(G-CLB-CLB) | NCT01010061 | open-label, phase 3 | 2013 | symptomatic Chronic lymphocytic leukaemia | Obinutuzumab+Chlorambucil | Chlorambucil | 241 | 116 |
| 52 | COMPLEMENT 1(RCLB-CLB) | NCT01010061 | open-label, phase 3 | 2013 | symptomatic Chronic lymphocytic leukaemia | Rituximab+Chlorambucil | Chlorambucil | 225 | 116 |
| 53 | COSMIC-311 | NCT03690388 | double-blind，phase phase 3 | 9/17/2021 | radioiodine-refractory differentiated thyroid cancer | Cabozantinib | Placebo | 125 | 62 |
| 54 | COU-AA-301 | NCT00638690 | double-blind, phase 3 | 4/28/2011 | Metastatic castration-resistant prostate cancer | Abiraterone acetate+Prednisone | Prednisone | 797 | 398 |
| 55 | COU-AA-302 | NCT00887198 | double-blind, phase 3 | 12/10/2012 | Metastatic castration-resistant prostate cancer | Abiraterone acetate+Prednisone | Prednisone | 546 | 542 |
| 56 | CROWN | NCT03052608 | open-label，phase phase 3 | 3/3/2021 | ALK-Positive Non–Small-Cell Lung Cancer | Lorlatinib | Crizotinib | 149 | 147 |
| 57 | CRYSTAL | NCT00154102 | open-label, phase 3 | 10/15/2021 | metastatic colorectal cancer | Cetuximab+FOLFIRI | FOLFIRI | 599 | 599 |
| 58 | DESTINY-Breast03 | NCT03529110 | open-label，phase phase 3 | 5/4/2022 | HER2-positive metastatic breast cancer | Trastuzumab deruxtecan | Trastuzumab emtansine | 261 | 263 |
| 59 | E1912 | NCT02048813 | open-label，phase phase 3 | 4/21/2020 | chronic lymphocytic leukemia | Ibrutinib–Rituximab Group | Chemoimmunotherapy Group | 354 | 175 |
| 60 | ECHELON-1 | NCT01712490 | open-label，phase phase 3 | 11/16/2018 | previously untreated stage III or IV classic Hodgkin’s lymphoma | brentuximab vedotin, doxorubicin, vinblastine, and dacarbazine | doxorubicin, bleomycin, vinblastine, and dacarbazine | 664 | 670 |
| 61 | ECHELON-2 | NCT01777152 | double-blind，phase phase 3 | 11/16/2018 | CD30-positive peripheral T-cell lymphomas | Brentuximab vedotin+cyclophosphamide+doxorubicin+prednisone | Vincristine+cyclophosphamide+doxorubicin+prednisone | 226 | 226 |
| 62 | ELEVATE-TN (A-O) | NCT02475681 | open-label, phase 3 | 11/21/2019 | chronic lymphocytic leukemia | Acalabrutinib monotherapy | Obinutuzumab chlorambucil | 179 | 177 |
| 62 | ELEVATE-TN (AO-O) | NCT02475681 | open-label, phase 3 | 11/21/2019 | chronic lymphocytic leukemia | Acalabrutinib obinutuzumab | Obinutuzumab chlorambucil | 179 | 177 |
| 63 | ELOQUENT-2 | NCT01239797 | open-label，phase 3 | 2015 | Relapsed or refractory multiple myeloma | Elotuzumab，lenalidomide and dexamethasone | Lenalidomide and dexamethasone | 321 | 325 |
| 64 | EMBRACA | NCT01945775 | open-label, phase 3 | 12/14/2018 | metastatic breast cancer with a germline BRCA1/2 mutation | Acalabrutinib monotherapy | Obinutuzumab chlorambucil | 179 | 177 |
| 65 | EMILIA | NCT00829166 | open-label, phase 3 | 2/22/2013 | metastatic HER2-positive breast cancer | Trastuzumab emtansine | Lapatinib+Capecitabine | 495 | 496 |
| 66 | EMPOWER-Lung1 | NCT03088540 | open-label，phase 3 | 2/22/2021 | aNSCLC | Cemiplimab | Chemotherapy | 283 | 280 |
| 67 | EMPOWER-Lung3 | NCT03409614 | double-blind，phase 3 | 11/8/2022 | Advanced NSCLC | Cemiplimab+Chemotherapy | Chemotherapy | 312 | 154 |
| 68 | ExteNET | NCT00878709 | double-blind，phase phase 3 | 7/17/2017 | HER2-positive breast cancer | Neratinib | Placebo | 1420 | 1420 |
| 69 | FLAURA | NCT02296125 | double-blind，phase phase 3 | 4/18/2018 | EGFR mutation–positive advanced non–small-cell lung cancer | Osimertinib | Standard EGFR-TKI | 279 | 277 |
| 70 | GADOLIN | NCT01059630 | open-label，phase phase 3 | 3/1/2016 | Rituximab-refractory indolent non-Hodgkin lymphoma | Obinutuzumab plus bendamustine | Bendamustine | 194 | 202 |
| 71 | GALLIUM | NCT01332968 | open-label, phase 3 | 12/17/2021 | untreated, advanced-stage CD20-positive follicular lymphoma | obinutuzumab+chemotherapy | Rituximab+chemotherapy | 601 | 601 |
| 72 | GO29365 | NCT02257567 | open-label, phase 2 | 6/10/2019 | diffuse large B-cell lymphoma | Polatuzumab vedotin+Bendamustine+Rituximab | Bendamustine+Rituximab | 40 | 40 |
| 73 | GOG 240 | NCT00803062 | open-label, phase 3 | 8/14/2014 | metastatic cervical cancer | Chemotherapy + Bevacizumab | Chemotherapy | 227 | 225 |
| 74 | GOG-0218 (BI-CON) | NCT00262847 | double-blind，phase phase 3 | 6/13/2018 | Ovarian Cancer | Chemotherapy with bevacizumab (throughout) | Chemotherapy | 625 | 625 |
| 74 | GOG-0218 (BT-CON) | NCT00262847 | double-blind，phase phase 3 | 6/13/2018 | Ovarian Cancer | Chemotherapy with bevacizumab (initiation) | Chemotherapy | 623 | 625 |
| 75 | HER2CLIMB | NCT02614794 | double-blind，phase phase 3 | 4/17/2020 | HER2-Positive Breast Cancer With Brain Metastases | Tucatinib | Placebo | 198 | 93 |
| 76 | HIMALAYA (DUR-SOR) | NCT03298451 | open-label, phase 3 | 10/24/2022 | Unresectable hepatocellular carcinoma | Durvalumab | Sorafenib | 389 | 389 |
| 76 | HIMALAYA (STR-SOR) | NCT03298451 | open-label, phase 3 | 10/24/2022 | Unresectable hepatocellular carcinoma | Tremelimumab+Durvalumab | Sorafenib | 393 | 389 |
| 77 | ICARIA-MM | NCT02990338 | open-label，phase phase 3 | 3/2/2020 | Relapsed and refractory multiple myeloma | Isatuximab，pomalidomide and dexamethasone | Pomalidomide plus dexamethasone | 154 | 153 |
| 78 | IKEMA | NCT03275285 | open-label，phase phase 3 | 3/31/2021 | Relapsed multiple myeloma | Isatuximab, pomalidomide and dexamethasone | Carfilzomib–dexamethasone | 179 | 123 |
| 79 | IMbrave150 | NCT03434379 | open-label，phase phase 3 | 5/29/2020 | Unresectable hepatocellular carcinoma | Atezolizumab and bevacizumab | Sorafenib | 336 | 165 |
| 80 | IMCgp100-202 | NCT03070392 | open-label，phase phase 3 | 1/25/2022 | Metastatic Uveal Melanoma | Tebentafusp | Pembrolizumab | 252 | 126 |
| 81 | IMpassion130 | NCT02425891 | double-blind，phase phase 3 | 3/8/2019 | Advanced Triple-Negative Breast Cancer | Atezolizumab plus nab-paclitaxel | Placebo plus nab-paclitaxel | 451 | 451 |
| 82 | IMpower010 | NCT02486718 | open-label，phase 3 | 10/15/2021 | Stage II-IIIA non-small-cell lung cancer | Atezolizumab | Best supportive care | 507 | 498 |
| 83 | IMpower110 | NCT02409342 | open-label，phase 3 | 5/18/2020 | non–small-cell lung cancer | Atezolizumab | Chemotherapy | 285 | 287 |
| 84 | IMpower133 | NCT02763579 | double-blind，phase 3 | 3/18/2019 | Extensive-Stage Small-Cell Lung Cancer | Atezolizumab plus carboplatin and etoposide | Carboplatin and etoposide | 201 | 202 |
| 85 | IMspire150 | NCT02908672 | double-blind，phase 3 | 7/30/2020 | Advanced BRAFV600 mutation positive melanoma | Atezolizumab, vemurafenib, and cobimetinib | Vemurafenib, and cobimetinib | 256 | 258 |
| 86 | INAVO120 | NCT04191499 | double-blind, phase 3 | 10/10/2024 | PIK3CA-mutated, hormone receptor–positive, HER2-negative metastatic breast cancer | Inavolisib+Palbociclib+Fulvestrant | Palbociclib+Fulvestrant | 161 | 164 |
| 87 | INO-VATEALL | NCT01564784 | open-label，phase 3 | 8/17/2017 | Acute Lymphoblastic Leukemia | Inotuzumab Ozogamicin | Sandard intensive chemotherapy | 164 | 162 |
| 88 | JAVELIN 100 | NCT02603432. | open-label, phase 3 | 6/30/2020 | Metastatic urothe lialcarcinom | Avelumab plus best supportive care | Best supportive care | 350 | 350 |
| 89 | JAVELIN 101 | NCT02684006 | open-label, phase 3 | 5/14/2019 | Advanced renal-cell carcinoma | Avelumab plus axitinib | Sunitinib | 442 | 444 |
| 90 | JUPITER-02 | NCT03581786 | double-blind, phase 3 | 10/30/2023 | metastatic nonkeratinizing nasopharyngeal carcinoma | Toripalimab+Gemcitabine+Cisplatin | Gemcitabine+Cisplatin | 146 | 143 |
| 91 | KATHERINE | NCT01772472 | open-label, phase 3 | 5/6/2019 | HER2-positive early breast cancer | Trastuzumab emtansine | Trastuzumab | 743 | 743 |
| 92 | KEYNOTE-006-PEMQ2IPI | NCT01866319 | open-label, phase 3 | 12/18/2015 | Unresectable stage III or stage IV melanoma | Pembrolizumab 10 mg/kg Q3W | Ipilimumab | 277 | 278 |
| 92 | KEYNOTE-006-PEMQ3IPI | NCT01866319 | open-label, phase 3 | 12/18/2015 | Unresectable stage III or stage IV melanoma | Pembrolizumab 10 mg/kg Q2W | Ipilimumab | 279 | 278 |
| 93 | KEYNOTE-024 | NCT02142738 | open-label, phase 3 | 4/11/2019 | Stage IV Non–Small-Cell Lung Cancer | Pembrolizumab | Chemotherapy | 154 | 151 |
| 94 | KEYNOTE-042 | NCT02220894 | open-label, phase 3 | 4/11/2019 | Advanced or metastatic non-small-cell lung cancer | Pembrolizumab | Chemotherapy | 637 | 637 |
| 95 | KEYNOTE-045 | NCT02256436 | open-label, phase 3 | 5/18/2017 | Advanced Urothelial Carcinoma | Pembrolizumab | Chemotherapy | 270 | 272 |
| 96 | KEYNOTE-048 (P-C) | NCT02358031 | open-label, phase 3 | 6/10/2019 | Recurrent or Metastatic Head and Neck Squamous Cell Carcinoma | Pembrolizumab monotherapy | Cetuximab-chemotherapy | 301 | 300 |
| 96 | KEYNOTE-048 (PC-C) | NCT02358031 | open-label, phase 3 | 6/10/2019 | Recurrent or Metastatic Head and Neck Squamous Cell Carcinoma | Pembrolizumab plus chemotherapy | Cetuximab plus chemotherapy | 281 | 300 |
| 97 | KEYNOTE-177 | NCT02563002 | open-label, phase 3 | 6/30/2020 | MSI-H/dMMR metastatic colorectal cancer | Pembrolizumab | chemotherapy | 153 | 154 |
| 98 | KEYNOTE-189 | NCT02578680 | double-blind, phase 3 | 8/20/2018 | Metastatic Non–Small-Cell Lung Cancer | Pembrolizumab plus chemotherapy | Chemotherapy | 410 | 206 |
| 99 | KEYNOTE-355 | NCT02819518 | double-blind, phase 3 | 11/13/2020 | metastatic triple-negative breast cancer | Pembrolizumab | Placebo | 566 | 281 |
| 100 | KEYNOTE-407 | NCT02775435 | double-blind, phase 3 | 10/30/2018 | Squamous Non–Small-Cell Lung Cancer | Pembrolizumab plus chemotherapy | Chemotherapy | 278 | 281 |
| 101 | KEYNOTE-426 | NCT02853331 | open-label, phase 3 | 4/19/2019 | Advanced Renal-Cell Carcinoma | Pembrolizumab plus axitinib | Sunitinib | 432 | 429 |
| 102 | KEYNOTE-522 | NCT03036488 | double-blind, phase 3 | 7/26/2021 | Triple-Negative Breast Cancer | Pembrolizumab plus Chemotherapy | Chemotherapy | 784 | 390 |
| 103 | KEYNOTE-564 | NCT03142334 | double-blind, phase 3 | 11/17/2021 | Renal-Cell Carcinoma | Pembrolizumab | Placebo | 496 | 498 |
| 104 | KEYNOTE-671 | NCT03425643 | double-blind, phase 3 | 10/16/2023 | stage II, IIIA, or IIIB (N2) non–small-cell lung cancer | Pembrolizumab | Placebo | 397 | 400 |
| 105 | KEYNOTE-716 | NCT03553836 | double-blind, phase 3 | 12/6/2021 | stage IIB or IIC cutaneous melanoma | Pembrolizumab | Placebo | 487 | 489 |
| 106 | KEYNOTE-775 | NCT03517449 | open-label, phase 3 | 7/21/2021 | Advanced Endometrial Cancer | Lenvatinib plus Pembrolizumab | Chemotherapy | 411 | 416 |
| 107 | KEYNOTE-826 | NCT03635567 | double-blind, phase 3 | 10/13/2021 | metastatic cervical cancer | Pembrolizumab | Placebo | 308 | 309 |
| 108 | KEYNOTE-A18 | NCT04221945 | double-blind, phase 3 | 1/12/2024 | locally advanced cervical cancer | Pembrolizumab+chemoradiotherapy | Chemoradiotherapy | 528 | 530 |
| 109 | LATITUDE | NCT01715285 | double-blind, phase 3 | 2/7/2018 | Castration-Sensitive Prostate Cancer | Androgen-deprivation therapy plus Abiraterone | Androgen-deprivation therapy | 597 | 602 |
| 110 | MAGNITUDE | NCT03748641 | double-blind, phase 3 | 8/11/2023 | Metastatic castration-resistant prostate cancer | Niraparib+abiraterone acetate+prednisone | abiraterone acetate+prednisone | 212 | 211 |
| 111 | MAIA | NCT02252172 | open-label, phase 3 | 6/27/2019 | Myeloma | Daratumumab plus lenalidomide and dexamethasone | Lenalidomide and dexamethasone alone | 368 | 369 |
| 112 | MARIPOSA | NCT04487080 | open-label, phase 3 | 8/20/2024 | EGFR-mutated metastatic non–small-cell lung cancer | Amivantamab+lazertinib | Osimertinib | 429 | 429 |
| 113 | METEOR | NCT01865747 | open-label, phase 3 | 5/5/2016 | Advanced Renal-Cell Carcinoma | Cabozantinib | Everolimus | 330 | 328 |
| 114 | METRIC | NCT01245062 | open-label, phase 3 | 2013 | Unresectable stage IIIC or IV cutaneous melanoma with BRAF V600 | Trametinib | chemotherapy | 214 | 108 |
| 115 | MONALEESA-7 | NCT02278120 | double-blind, phase 3 | 7/18/2018 | Hormone-receptor-positive advanced breast cancer | Ribociclib plus tamoxifen or NSAI | Tamoxifen or NSAI | 335 | 337 |
| 116 | MONARCH 2 | NCT02107703 | double-blind, phase 3 | 9/28/2017 | HR+/HER2- Advanced Breast Cancer | Abemaciclib + Fulvestrant | Fulvestrant | 446 | 223 |
| 117 | MONARCH 3 | NCT02246621 | double-blind, phase 3 | 2/26/2018 | HR-positive, HER2-negative advanced breast cancer | Abemaciclib+nonsteroidal aromatase inhibitor | nonsteroidal aromatase inhibitor | 328 | 165 |
| 118 | monarchE | NCT03155997 | open-label, phase 3 | 10/12/2021 | HR+/HER2- early breast cancer | Abemaciclib plus endocrine therapy | Endocrine therapy | 2808 | 2829 |
| 119 | MPACT | NCT00844649 | open-label, phase 3 | 9/6/2013 | Metastatic pancreatic adenocarcinoma | nab-Paclitaxel+gemcitabine | Gemcitabine | 431 | 430 |
| 120 | MURANO | NCT02005471 | open-label, phase 3 | 6/8/2018 | Chronic Lymphocytic Leukemia | Venetoclax plus rituximab | Bendamustine plus rituximab | 194 | 195 |
| 121 | NAPOLI-1 (N-F) | NCT01494506 | open-label, phase 3 | 10/23/2015 | metastatic pancreatic cancer | Nanoliposomal irinotecan plus fl uorouracil and folinic acid combination therapy | Fluorouracil and folinic acid  combination therapy  control | 117 | 119 |
| 121 | NAPOLI-1 (NF-F) | NCT01494506 | open-label, phase 3 | 10/23/2015 | metastatic pancreatic cancer | Nanoliposomal irinotecan monotherapy | Fluorouracil and folinic acid  combination therapy  control | 151 | 149 |
| 122 | NATALEE | NCT03701334 | open-label, phase 3 | 9/17/2024 | HR-positive, HER2-negative early breast cancer | Ribociclib+nonsteroidal aromatase inhibitor | Nonsteroidal aromatase inhibitor | 2549 | 2552 |
| 123 | NCT01343277 | NCT01343277 | open-label, phase 3 | 10/23/2015 | advanced liposarcoma or leiomyosarcoma | Trabectedin | Dacarbazine | 345 | 173 |
| 124 | NCT01539512 | NCT01539512 | double-blind, phase 3 | 7/24/2014 | Relapsed Chronic Lymphocytic Leukemia | Idelalisib+Rituximab | Rituximab | 110 | 110 |
| 125 | NETTER-1 | NCT01578239 | open-label，phase 3 | 4/23/2024 | metastatic midgut neuroendocrine tumors | 177Lu-Dotatateplus+octreotide long-acting repeatable | Octreotide long-acting repeatable | 116 | 113 |
| 126 | NSABP B31 and NCCTG N9831 | NCT00004067 | open-label, phase 3 | 11/20/2006 | HER2-positive breast cancer | Doxorubicin+Cyclophosphamide → Paclitaxel+Trastuzumab | Doxorubicin+Cyclophosphamide → Paclitaxel | 1672 | 1679 |
| 127 | OAK | NCT02008227 | open-label，phase 3 | 10/18/2016 | previously treated non-small-cell lung cancer | Atezolizumab | Docetaxel | 609 | 578 |
| 128 | OlympiAD | NCT02000622 | open-label，phase 3 | 1/12/2018 | metastatic breast cancer and a germline BRCA mutation | Olaparib | Chemotherapy | 205 | 97 |
| 129 | OPTiM | NCT00769704 | open-label, phase 3 | 10/27/2015 | Advanced Melanoma | Intralesional Talimogene laherparepvec | Granulocyte macrophage colony-stimulating factor | 295 | 141 |
| 130 | PACIFIC | NCT02125461 | double-blind，phase 3 | 2/16/2018 | stage III, unresectable non–small-cell lung cancer | Durvalumab | Placebo | 473 | 236 |
| 131 | PALOMA3 | NCT01942135 | double-blind, phase 3 | 3/31/2017 | HR-positive, HER2-negative early breast cancer | Palbociclib+Fulvestrant | Fulvestrant | 347 | 174 |
| 132 | PANORAMA | NCT01023308 | double-blind, phase 3 | 2015 | refractory multiple myeloma | Panobinostat+Bortezomib+Dexamethasone | Bortezomib+Dexamethasone | 387 | 381 |
| 133 | PAOLA-1 | NCT02477644 | double-blind，phase 3 | 5/8/2020 | newly diagnosed, advanced, high-grade ovarian cancer and were having a response after first-line platinum–taxane chemotherapy plus bevacizumab | Olaparib plus Bevacizumab | Bevacizumab | 537 | 269 |
| 134 | PARAMOUNT | NCT00789373 | double-blind, phase 3 | 2012 | Advanced non-squamous non-small-cell lung cancer | Pemetrexed+best supportive care | Best supportive care | 359 | 180 |
| 135 | POLLUX | NCT02076009 | open-label，phase 3 | 11/21/2016 | Multiple Myeloma | Daratumumab, lenalidomide and dexamethasone | Lenalidomide and dexamethasone | 286 | 283 |
| 136 | POLO | NCT02184195 | double-blind，phase 3 | 12/27/2019 | BRCA-Mutated Metastatic Pancreatic Cancer | Olaparib | Placebo | 92 | 62 |
| 137 | POPLAR | NCT01903993 | open-label，2 | 10/18/2016 | previously treated non-small-cell lung cancer | Atezolizumab | Docetaxel | 144 | 143 |
| 138 | POSEIDON | NCT03164616 | open-label, phase 3 | 11/18/2022 | metastatic (Stage IV) non-small-cell lung cancer (mNSCLC) with EGFR/ALK wild-type | Durvalumab+chemotherapy | Chemotherapy | 338 | 337 |
| 139 | PRIMA | NCT00140582 | open-label, phase 3 | 4/29/2020 | untreated follicular lymphoma | Rituximab | Observation | 505 | 513 |
| 140 | PROFILE 1014 | NCT01526928 | open-label, phase 3 | 3/11/2016 | Previously untreated advanced non–small-cell lung cancer (NSCLC) with ALK | Crizotinib | Chemotherapy | 172 | 171 |
| 141 | PROLONG | NCT01039376 | open-label, phase 3 | 1/19/2016 | chronic lymphocytic leukemia | Ofatumumab | Observation | 238 | 236 |
| 142 | PSMAfore | NCT04689828 | open-label, phase 3 | 3/28/2025 | PSMA-positive metastatic castration-resistant prostate cancer | [177Lu]Lu-PSMA-617 | abiraterone or enzalutamide | 234 | 234 |
| 143 | RADIANT-3 | NCT00510068 | double-blind, phase 3 | 2/26/2016 | Advanced (unresectable or metastatic) low‑grade or intermediate‑grade pancreatic neuroendocrine tumors | Everolimus | placebo | 207 | 203 |
| 144 | REACH3 | NCT03112603 | open-label, phase 3 | 2021 | 不是肿瘤 |  |  |  |  |
| 145 | RECORD-1 | NCT00410124 | double-blind, phase 3 | 2009 | Metastatic renal cell carcinoma | Everolimus+best supportive care | best supportive care | 272 | 138 |
| 146 | RELATIVITY-047 | NCT03470922 | double-blind, phase2-3 | 3/21/2022 | unresectable melanoma (unresectable stage III or stage IV) | Relatlimab+nivolumab | Nivolumab | 355 | 359 |
| 147 | RELAY | NCT02411448 | double-blind, phase2-3 | 5/29/2020 | stage IV (metastatic) non-small-cell lung cancer | Ramucirumab+erlotinib | Erlotinib | 224 | 225 |
| 148 | RUBY | NCT03981796 | double-blind, phase2-3 | 8/1/2014 | advanced (FIGO stage III or IV) or first recurrent endometrial cancer | Dostarlimab+carboplatin | Carboplatin | 245 | 249 |
| 149 | SEQUOIA | NCT03336333 | open-label, phase 3 | 1/19/2023 | Untreated chronic lymphocytic leukaemia | Zanubrutinib | Bendamustine+rituximab | 240 | 227 |
| 150 | SOLO-1 | NCT01844986 | double-blind，phase 3 | 12/19/2018 | Newly Diagnosed Advanced Ovarian Cancer | Olaparib | Placebo | 260 | 131 |
| 151 | SOPHIA | NCT02492711 | open-label，phase 3 | 12/16/2020 | ERRB2 (formerly HER2)–positive advanced breast cancer | Margetuximab | Trastuzumab | 266 | 270 |
| 152 | SPARTAN | NCT01946204 | double-blind，phase 3 | 2/14/2018 | metastatic, castration-sensitive prostate cancer | Apalutamide | Placebo | 806 | 401 |
| 153 | SSGXVIII | NCT00116935 | open-label, phase 3 | 1/31/2012 | KIT‑positive gastrointestinal stromal tumor | imatinib 36Months | imatinib 12Months | 198 | 199 |
| 154 | S-TRAC | NCT00375674 | double-blind, phase2-3 | 11/16/2017 | Locoregional clear-cell renal-cell carcinoma | Sunitinib | placebo | 309 | 306 |
| 155 | SUN 111 | NCT00428597 | double-blind, phase2-3 | 2011 | advanced pancreatic neuroendocrine tumors | Sunitinib | best supportive care | 86 | 85 |
| 156 | TALAPRO-2 | NCT03395197 | double-blind, phase 3 | 6/20/2023 | metastatic castration-resistant prostate cancer | Talazoparib+enzalutamide | enzalutamide | 402 | 403 |
| 157 | TAX 324 | NCT00273546 | open-label, phase 3 | 2007 | stage III or IV squamous cell carcinoma of the head and neck | docetaxel+cisplatin+fluorouracil | cisplatin+fluorouracil | 255 | 246 |
| 158 | TITAN | NCT02489318 | double-blind，phase 3 | 9/17/2019 | Metastatic,Castration-Sensitive Prostate Cancer | Apalutamide to androgen-deprivation therapy | Androgen-deprivation therapy | 525 | 527 |
| 159 | TIVO-3 | NCT02627963 | open-label，phase 3 | 3/10/2021 | Metastatic renal cell carcinoma | Tivozanib | Sorafenib | 175 | 175 |
| 160 | TOPAZ-1 | NCT03875235 | double-blind, phase 3 | 9/2/2022 | metastatic biliary tract cancer | Durvalumab+gemcitabine+cisplatin | gemcitabine+cisplatin | 341 | 344 |
| 161 | TRANSFORM | NCT03575351 | open-label，phase 3 | 6/24/2022 | Relapsed or refractory large B-cell lymphoma | Lisocabtagene maraleucel | Immunochemotherapy | 92 | 92 |
| 162 | TROPiCS-02 | NCT03901339 | open-label，phase 3 | 2/3/2023 | HER2-Negative Metastatic Breast Cancer | Sacituzumab govitecan | chemotherapy | 272 | 271 |
| 163 | VIALE-A | NCT02993523 | double-blind, phase 3 | 11/21/2018 | untreated acute myeloid leukemia | Azacitidine+venetoclax | Azacitidine | 286 | 145 |
| 164 | VISTA | NCT00111319 | open-label, phase 3 | 2014 | Newly diagnosed, symptomatic multiple myeloma | Bortezomib+melphalan+prednisone | Melphalan+prednisone | 344 | 338 |
| 165 | ZUMA-7 | NCT03391466 | open-label，phase 3 | 4/1/2022 | Large B-Cell Lymphoma | Axicabtagene ciloleucel | chemoimmunotherapy | 180 | 179 |

# Table S4 Overall and Subgroup Meta-analysis of RMST ratio for OS before and after data updates

| Variable | Subgroup | No. of studies | Pooled initial RMST ratio (95% CI) | Pooled updated RMST ratio (95% CI) | Comparisons with increased RMST ratios | Comparisons with significantly increased RMST ratios | I²  (initial reports) | I² (updated reports) |
| --- | --- | --- | --- | --- | --- | --- | --- | --- |
| Overall | All Studies | 150 | 1.08 (1.07–1.10) | 1.13 (1.11–1.14) | 123 | 7 | 87 | 90.2 |
| Number of Patients | Large(>600) | 60 | 1.07 (1.05–1.08) | 1.11 (1.09–1.13) | 53 | 6 | 91.2 | 94.8 |
| Number of Patients | Medium (100-600) | 89 | 1.10 (1.08–1.12) | 1.14 (1.12–1.17) | 69 | 1 | 67.7 | 65.9 |
| FDA Approve Year | Pre-2015 | 29 | 1.09 (1.07–1.11) | 1.13 (1.10–1.17) | 24 | 3 | 68.2 | 77.1 |
| FDA Approve Year | 2016–2020 | 75 | 1.08 (1.07–1.10) | 1.13 (1.10–1.15) | 66 | 2 | 83.7 | 85.2 |
| FDA Approve Year | Post-2020 | 46 | 1.09 (1.06–1.11) | 1.13 (1.09–1.16) | 33 | 2 | 92 | 93.8 |
| 5-year survival of indication | Intermediate (30%-69%) | 85 | 1.09 (1.08–1.11) | 1.13 (1.11–1.15) | 69 | 3 | 77.2 | 71.9 |
| 5-year survival of indication | High survival (70%-1) | 23 | 1.03 (1.02–1.04) | 1.06 (1.03–1.08) | 17 | 3 | 74.1 | 95.1 |
| 5-year survival of indication | Low survival (<30%) | 42 | 1.11 (1.08–1.13) | 1.17 (1.14–1.21) | 37 | 1 | 64.6 | 71.8 |
| PH Assumption | PH | 110 | 1.07 (1.06–1.08) | 1.11 (1.09–1.12) | 88 | 6 | 85.1 | 88.5 |
| PH Assumption | Non-PH | 40 | 1.12 (1.09–1.15) | 1.19 (1.14–1.23) | 35 | 1 | 76.5 | 79.6 |
| Treatment Line | First-line | 89 | 1.07 (1.06–1.09) | 1.12 (1.10–1.14) | 76 | 5 | 88.4 | 93.4 |
| Treatment Line | Subsequent Line | 61 | 1.10 (1.08–1.13) | 1.14 (1.11–1.17) | 47 | 2 | 73.3 | 67.6 |
| Treatement comparisons | Targeted VS. Supportive care/Placebo | 10 | 1.07 (1.02–1.13) | 1.07 (1.02–1.12) | 6 | 0 | 72.3 | 54.9 |
| Treatement comparisons | Targeted VS. Chemotherapy | 9 | 1.16 (1.06–1.28) | 1.20 (1.08–1.33) | 7 | 0 | 76.8 | 66.7 |
| Treatement comparisons | Targeted-Chemotherapy VS. Chemotherapy | 11 | 1.14 (1.05–1.24) | 1.20 (1.09–1.33) | 10 | 1 | 91.6 | 92.4 |
| Treatement comparisons | Targeted VS. Targeted | 16 | 1.05 (1.03–1.06) | 1.08 (1.05–1.11) | 13 | 0 | 25 | 35.1 |
| Treatement comparisons | Others | 27 | 1.08 (1.05–1.11) | 1.13 (1.09–1.18) | 23 | 3 | 88.6 | 92.6 |
| Treatement comparisons | Hormonal VS. Hormonal | 6 | 1.06 (1.03–1.10) | 1.10 (1.07–1.13) | 6 | 1 | 80.1 | 48.4 |
| Treatement comparisons | Immunotherapy VS. Chemotherapy | 13 | 1.17 (1.14–1.21) | 1.26 (1.21–1.31) | 13 | 0 | 0 | 0 |
| Treatement comparisons | Immuno-Chemotherapy VS. Chemotherapy | 18 | 1.09 (1.07–1.12) | 1.15 (1.11–1.19) | 17 | 1 | 55 | 65.8 |
| Treatement comparisons | Immuno-Targeted VS. Targeted | 8 | 1.06 (1.04–1.09) | 1.10 (1.07–1.13) | 8 | 1 | 43.5 | 0 |
| Treatement comparisons | Immunotherapy VS. Targeted | 4 | 1.12 (1.08–1.17) | 1.19 (1.13–1.26) | 4 | 0 | 0 | 0 |
| Treatement comparisons | Immuno-Targeted VS. Immuno-Chemotherapy | 4 | 1.03 (1.00–1.05) | 1.05 (1.02–1.07) | 3 | 0 | 37.1 | 0.1 |
| Treatement comparisons | Chemotherapy VS. Chemotherapy | 6 | 1.19 (1.09–1.29) | 1.19 (1.10–1.29) | 3 | 0 | 39.2 | 25.5 |
| Treatement comparisons | Immunotherapy VS. Immunotherapy | 4 | 1.13 (1.05–1.22) | 1.21 (1.13–1.30) | 3 | 0 | 54.2 | 0 |
| Treatement comparisons | Immunotherapy VS. Supportive care/Placebo | 8 | 1.07 (1.02–1.11) | 1.06 (1.01–1.11) | 4 | 0 | 81.4 | 82.8 |
| Treatement comparisons | Targeted-Hormonal VS. Hormonal | 6 | 1.01 (1.00–1.01) | 1.03 (1.00–1.06) | 3 | 0 | 0 | 90.9 |
| Blinding | Yes | 51 | 1.06 (1.05–1.08) | 1.10 (1.08–1.12) | 45 | 2 | 78.2 | 77.8 |
| Blinding | No | 99 | 1.10 (1.08–1.11) | 1.14 (1.12–1.17) | 78 | 5 | 87.7 | 91 |
| Phase | 3 | 146 | 1.08 (1.07–1.09) | 1.13 (1.11–1.14) | 119 | 7 | 87 | 90.3 |
| Phase | 2 | 4 | 1.30 (1.05–1.63) | 1.42 (1.07–1.89) | 4 | 0 | 76.4 | 70.4 |
| Crossover | Yes | 59 | 1.09 (1.07–1.10) | 1.13 (1.10–1.16) | 48 | 4 | 83.6 | 85.5 |
| Crossover | No | 91 | 1.08 (1.07–1.10) | 1.13 (1.10–1.15) | 75 | 3 | 87.7 | 90.9 |
| Disease | Others | 29 | 1.11 (1.07–1.14) | 1.14 (1.10–1.17) | 23 | 0 | 73.8 | 64.7 |
| Disease | Leukemia | 18 | 1.11 (1.05–1.16) | 1.15 (1.07–1.23) | 14 | 0 | 91.5 | 91.7 |
| Disease | Lung Cancer | 25 | 1.10 (1.07–1.13) | 1.18 (1.13–1.23) | 24 | 1 | 63.7 | 74 |
| Disease | Breast Cancer | 18 | 1.07 (1.03–1.10) | 1.09 (1.05–1.14) | 13 | 2 | 97.7 | 98.3 |
| Disease | Prostate Cancer | 12 | 1.05 (1.03–1.07) | 1.08 (1.06–1.10) | 10 | 1 | 64.4 | 34.3 |
| Disease | Lymphoma | 8 | 1.06 (1.02–1.09) | 1.06 (1.02–1.11) | 6 | 0 | 45.1 | 60.9 |
| Disease | Colorectal Cancer | 7 | 1.15 (1.06–1.25) | 1.22 (1.11–1.34) | 6 | 0 | 65.5 | 69.1 |
| Disease | Head and Neck Cancer | 5 | 1.18 (1.11–1.25) | 1.23 (1.15–1.31) | 4 | 0 | 0 | 0 |
| Disease | Kidney Cancer | 12 | 1.07 (1.04–1.10) | 1.09 (1.06–1.13) | 10 | 0 | 57 | 57.2 |
| Disease | Multiple Myeloma | 6 | 1.03 (1.02–1.05) | 1.15 (1.03–1.28) | 6 | 3 | 0.1 | 91.1 |
| Disease | Melanoma | 10 | 1.11 (1.07–1.16) | 1.16 (1.11–1.22) | 7 | 0 | 51.6 | 0 |
| Metastatic | No | 36 | 1.03 (1.02–1.04) | 1.05 (1.04–1.07) | 28 | 3 | 73.9 | 90.3 |
| Metastatic | Yes | 114 | 1.11 (1.09–1.12) | 1.16 (1.14–1.18) | 95 | 4 | 68.8 | 67.1 |
| Update time duration | Medium (12-24 months) | 41 | 1.11 (1.08–1.13) | 1.16 (1.12–1.20) | 35 | 1 | 71.9 | 81.5 |
| Update time duration | Short (<12 months) | 47 | 1.10 (1.07–1.12) | 1.12 (1.10–1.15) | 36 | 0 | 87.3 | 90.2 |
| Update time duration | Long (>24 months) | 62 | 1.06 (1.05–1.08) | 1.11 (1.09–1.13) | 52 | 6 | 85.5 | 87.9 |
| Information fraction of initial OS | Immature (<50%) | 58 | 1.04 (1.03–1.05) | 1.07 (1.05–1.09) | 43 | 5 | 79.6 | 91.9 |
| Information fraction of initial OS | Moderate (50%-75%) | 35 | 1.14 (1.11–1.17) | 1.20 (1.16–1.24) | 31 | 2 | 66.8 | 55.2 |
| Information fraction of initial OS | Mature (>75%) | 57 | 1.11 (1.09–1.13) | 1.15 (1.12–1.18) | 49 | 0 | 55.3 | 63.9 |

Note, The grouping criteria for each subgroup are described in Table S2; Subgroups with fewer than two studies were excluded from quantitative analyses. Both the RMST ratio and OR represent comparisons of the experimental group versus the control group; OS, overall survival

# Table S5 Overall and Subgroup Meta-analysis of RMST ratio for PFS/EFS before and after data updates

| Variable | Subgroup | No. of  studies | Pooled initial RMST ratio (95% CI) | Pooled updated RMST ratio (95% CI) | Comparisons with increased RMST ratios | Comparisons with significantly increased RMST ratios | I² (initial reports) | I² (updated reports) |
| --- | --- | --- | --- | --- | --- | --- | --- | --- |
| Overall | All Studies | 120 | 1.28 (1.24–1.31) | 1.42 (1.37–1.48) | 111 | 24 | 96.3 | 96.9 |
| Number of Patients | Large(>600) | 53 | 1.20 (1.15–1.25) | 1.30 (1.24–1.36) | 49 | 7 | 97.9 | 97.9 |
| Number of Patients | Medium (100-600) | 66 | 1.34 (1.30–1.39) | 1.54 (1.47–1.62) | 62 | 17 | 81.1 | 81.7 |
| FDA Approve Year | Pre-2015 | 20 | 1.33 (1.24–1.42) | 1.43 (1.32–1.55) | 15 | 2 | 87.5 | 87.9 |
| FDA Approve Year | 2016–2020 | 62 | 1.26 (1.21–1.31) | 1.42 (1.35–1.49) | 60 | 14 | 95.5 | 95.3 |
| FDA Approve Year | Post-2020 | 38 | 1.28 (1.21–1.35) | 1.43 (1.32–1.55) | 36 | 8 | 96.9 | 98.1 |
| 5-year survival of indication | Intermediate (30%-69%) | 76 | 1.27 (1.23–1.32) | 1.40 (1.34–1.46) | 69 | 10 | 91.5 | 91.4 |
| 5-year survival of indication | Low survival (<30%) | 27 | 1.34 (1.27–1.42) | 1.53 (1.41–1.67) | 26 | 6 | 81.5 | 87.7 |
| 5-year survival of indication | High survival (70%-) | 17 | 1.20 (1.10–1.31) | 1.36 (1.22–1.51) | 16 | 8 | 99.4 | 99.5 |
| PH Assumption | PH | 62 | 1.29 (1.24–1.34) | 1.45 (1.37–1.53) | 56 | 9 | 92.3 | 94.2 |
| PH Assumption | Non-PH | 58 | 1.26 (1.21–1.31) | 1.40 (1.33–1.48) | 55 | 15 | 97.3 | 97.6 |
| Treatment Line | First-line | 74 | 1.22 (1.18–1.26) | 1.36 (1.30–1.43) | 71 | 17 | 95.8 | 97.6 |
| Treatment Line | Subsequent Line | 46 | 1.38 (1.31–1.45) | 1.53 (1.45–1.62) | 40 | 7 | 90.4 | 87.5 |
| Treatement comparisons | Targeted VS. Supportive care/Placebo | 10 | 1.41 (1.26–1.57) | 1.53 (1.31–1.79) | 9 | 2 | 95.1 | 96.7 |
| Treatement comparisons | Targeted VS. Chemotherapy | 5 | 1.73 (1.59–1.89) | 1.93 (1.67–2.24) | 4 | 0 | 0 | 30 |
| Treatement comparisons | Targeted VS. Targeted | 14 | 1.29 (1.19–1.39) | 1.49 (1.31–1.70) | 14 | 3 | 87.1 | 90.5 |
| Treatement comparisons | Others | 28 | 1.25 (1.18–1.33) | 1.35 (1.27–1.44) | 23 | 4 | 94.8 | 93.5 |
| Treatement comparisons | Immunotherapy VS. Chemotherapy | 11 | 1.24 (1.12–1.37) | 1.47 (1.26–1.71) | 11 | 2 | 78.5 | 84.6 |
| Treatement comparisons | Targeted-Hormonal VS. Hormonal | 9 | 1.18 (1.04–1.35) | 1.32 (1.15–1.50) | 7 | 3 | 99.1 | 99.1 |
| Treatement comparisons | Immuno-Targeted VS. Targeted | 8 | 1.27 (1.18–1.38) | 1.41 (1.28–1.55) | 8 | 2 | 78.9 | 76.6 |
| Treatement comparisons | Immuno-Chemotherapy VS. Chemotherapy | 12 | 1.27 (1.18–1.35) | 1.40 (1.26–1.56) | 12 | 2 | 80.8 | 88.4 |
| Treatement comparisons | Immunotherapy VS. Immunotherapy | 4 | 1.27 (1.16–1.39) | 1.50 (1.21–1.85) | 4 | 2 | 60.7 | 82.5 |
| Treatement comparisons | Immunotherapy VS. Supportive care/Placebo | 11 | 1.24 (1.16–1.32) | 1.35 (1.24–1.48) | 11 | 0 | 82.2 | 85.6 |
| Treatement comparisons | Immuno-Targeted VS. Immuno-Chemotherapy | 4 | 1.29 (1.11–1.49) | 1.62 (1.30–2.01) | 4 | 4 | 94.7 | 94.6 |
| Treatement comparisons | Targeted-Chemotherapy VS. Chemotherapy | 4 | 1.10 (1.07–1.12) | 1.16 (1.06–1.27) | 4 | 0 | 0 | 85.6 |
| Blinding | Yes | 44 | 1.28 (1.22–1.33) | 1.39 (1.32–1.47) | 40 | 6 | 96.6 | 95.9 |
| Blinding | No | 76 | 1.28 (1.23–1.32) | 1.45 (1.37–1.52) | 71 | 18 | 95 | 96.6 |
| Disease | Others | 29 | 1.27 (1.21–1.33) | 1.39 (1.29–1.49) | 26 | 3 | 77.8 | 87.4 |
| Disease | Lung Cancer | 20 | 1.34 (1.27–1.41) | 1.57 (1.42–1.73) | 20 | 6 | 71.2 | 86.5 |
| Disease | Lymphoma | 11 | 1.35 (1.19–1.54) | 1.43 (1.23–1.65) | 9 | 0 | 94.2 | 93.7 |
| Disease | Breast Cancer | 19 | 1.25 (1.15–1.36) | 1.34 (1.21–1.48) | 18 | 2 | 99.5 | 99.5 |
| Disease | Melanoma | 15 | 1.24 (1.16–1.32) | 1.39 (1.28–1.51) | 14 | 3 | 83.7 | 76.2 |
| Disease | Multiple Myeloma | 8 | 1.14 (1.03–1.26) | 1.30 (1.18–1.42) | 7 | 3 | 92.5 | 85.2 |
| Disease | Kidney Cancer | 8 | 1.28 (1.14–1.43) | 1.40 (1.25–1.57) | 8 | 1 | 90.8 | 86.4 |
| Disease | Leukemia | 10 | 1.36 (1.22–1.52) | 1.60 (1.42–1.81) | 9 | 6 | 94.1 | 90.9 |
| Metastatic | No | 27 | 1.17 (1.11–1.22) | 1.30 (1.21–1.39) | 26 | 9 | 98 | 98.8 |
| Metastatic | Yes | 93 | 1.31 (1.27–1.36) | 1.47 (1.41–1.53) | 85 | 15 | 90.6 | 90.4 |
| Update time duration | Long (>24 months) | 49 | 1.22 (1.18–1.27) | 1.41 (1.32–1.50) | 47 | 15 | 94 | 96.6 |
| Update time duration | Medium (12-24 months) | 41 | 1.29 (1.23–1.36) | 1.43 (1.34–1.52) | 38 | 6 | 95.5 | 96 |
| Update time duration | Short (<12 months) | 30 | 1.36 (1.27–1.45) | 1.45 (1.34–1.56) | 26 | 3 | 91.8 | 92.2 |
| Information fraction of initial PFS/EFS | Immature (<50%) | 11 | 1.19 (1.11–1.27) | 1.31 (1.17–1.47) | 10 | 3 | 90.5 | 93.9 |
| Information fraction of initial PFS/EFS | Moderate (50%-75%) | 47 | 1.31 (1.25–1.37) | 1.49 (1.40–1.59) | 42 | 15 | 97.4 | 97.3 |
| Information fraction of initial PFS/EFS | Mature (>75%) | 62 | 1.27 (1.22–1.32) | 1.39 (1.33–1.46) | 59 | 6 | 93.5 | 95.1 |

Note, The grouping criteria for each subgroup are described in Table S2; Subgroups with fewer than two studies were excluded from quantitative analyses. Both the RMST ratio and OR represent comparisons of the experimental group versus the control group; PFS, and EFS refer to progression-free survival, and event-free (including disease-free or relapse-free) survival, respectively

# Table S6 The distribution of trial-specific τ values for OS

| Trial | NCT | Initial τ/month | Updated τ/month |
| --- | --- | --- | --- |
| ACOSOG Z9001 | NCT00041197 | 48.100 | 69.400 |
| ADMIRAL | NCT02421939 | 28.000 | 42.653 |
| AG120-C-005 | NCT02989857 | 15.296 | 22.005 |
| AG120-C-009 | NCT03173248 | 29.750 | 46.200 |
| ALEX | NCT02075840 | 25.000 | 57.952 |
| ALFA-0701 | NCT00927498 | 38.000 | 53.719 |
| ALTA-1 | NCT02737501 | 16.545 | 26.856 |
| APHINITY | NCT01358877 | 71.500 | 99.712 |
| ARAMIS | NCT02200614 | 42.000 | 52.178 |
| ARCHES | NCT02677896 | 22.689 | 52.415 |
| ASCENT | NCT02574455 | 23.700 | 27.300 |
| ATTRACTION-3 | NCT02569242 | 33.900 | 52.200 |
| AUGMENT | NCT01938001 | 44.700 | 90.600 |
| BEACONCRC (DOU-CON) | NCT02928224 | 17.000 | 21.877 |
| BEACONCRC (TRI-CON) | NCT02928224 | 16.000 | 21.877 |
| Bonner | NCT00004227 | 59.500 | 66.900 |
| BREAKWATER | NCT04607421 | 23.000 | 35.300 |
| BRIGHT AML 1003 | NCT01546038 | 20.085 | 36.489 |
| CABOSUN | NCT01835158 | 29.445 | 36.152 |
| CASPIAN | NCT03043872 | 21.291 | 27.247 |
| CASSIOPEIA | NCT02541383 | 28.741 | 94.229 |
| CASTOR | NCT02136134 | 13.550 | 77.300 |
| CheckMate 816 | NCT02998528 | 32.100 | 83.300 |
| CheckMate 9LA | NCT03215706 | 16.200 | 82.600 |
| CheckMate025 | NCT01668784 | 28.846 | 76.818 |
| CheckMate057 | NCT01673867 | 23.729 | 33.000 |
| CheckMate141 | NCT02105636 | 13.577 | 29.223 |
| CheckMate214 | NCT02231749 | 29.739 | 40.843 |
| CheckMate227 | NCT02477826 | 42.685 | 71.040 |
| CheckMate649 | NCT02872116 | 37.317 | 44.906 |
| CheckMate743 | NCT02899299 | 37.049 | 48.318 |
| CheckMate9ER | NCT03141177 | 27.000 | 41.450 |
| CLEAR | NCT02811861 | 36.739 | 44.434 |
| CLEOPATRA | NCT00567190 | 37.700 | 120.000 |
| CLL | NCT02242942 | 34.958 | 73.000 |
| CLL8 | NCT00281918 | 60.950 | 96.100 |
| CLTR0310-301 | NCT01696084 | 29.000 | 61.993 |
| coBRIM | NCT01689519 | 13.000 | 72.000 |
| CodeBreaK 300 (240-C) | NCT05198934 | 12.998 | 19.596 |
| CodeBreaK 300 (960-C) | NCT05198934 | 12.998 | 19.785 |
| COLUMBUS (ENB-EN) | NCT01909453 | 43.000 | 100.500 |
| COLUMBUS (ENB-VEM) | NCT01909453 | 43.000 | 103.504 |
| COMBI-d | NCT01584648 | 15.800 | 37.900 |
| COMPLEMENT 1(G-CLB-CLB) | NCT01010061 | 34.579 | 44.145 |
| COMPLEMENT 1(RCLB-CLB) | NCT01010061 | 34.579 | 44.242 |
| COSMIC-311 | NCT03690388 | 7.065 | 11.207 |
| COU-AA-301 | NCT00638690 | 16.900 | 25.100 |
| COU-AA-302 | NCT00887198 | 34.700 | 56.900 |
| CRYSTAL | NCT00154102 | 34.300 | 53.800 |
| DESTINY-Breast03 | NCT03529110 | 22.000 | 59.392 |
| E1912 | NCT02048813 | 49.000 | 84.000 |
| ECHELON-2 | NCT01777152 | 59.066 | 81.688 |
| ELEVATE-TN (A-O) | NCT02475681 | 40.800 | 88.749 |
| ELEVATE-TN (AO-O) | NCT02475681 | 40.800 | 88.749 |
| EMBRACA | NCT01945775 | 33.400 | 57.700 |
| EMILIA | NCT00829166 | 35.700 | 67.800 |
| EMPOWER-Lung1 | NCT03088540 | 27.432 | 37.550 |
| EMPOWER-Lung3 | NCT03409614 | 22.652 | 29.261 |
| FLAURA | NCT02296125 | 25.485 | 48.386 |
| GADOLIN | NCT01059630 | 42.700 | 52.741 |
| GALLIUM | NCT01332968 | 52.300 | 115.500 |
| GO29365 | NCT02257567 | 25.100 | 50.400 |
| GOG 240 | NCT00803062 | 33.800 | 51.098 |
| GOG-0218 (BI-CON) | NCT00262847 | 46.256 | 124.456 |
| GOG-0218 (BT-CON) | NCT00262847 | 46.258 | 123.183 |
| HER2CLIMB | NCT02614794 | 22.759 | 28.157 |
| HIMALAYA (DUR-SOR) | NCT03298451 | 43.539 | 59.595 |
| HIMALAYA (STR-SOR) | NCT03298451 | 42.861 | 59.595 |
| ICARIA-MM | NCT02990338 | 16.000 | 36.217 |
| IMbrave150 | NCT03434379 | 15.097 | 26.293 |
| IMCgp100-202 | NCT03070392 | 27.708 | 50.014 |
| IMpassion130 | NCT02425891 | 30.333 | 35.128 |
| IMpower010 | NCT02486718 | 56.596 | 63.389 |
| IMpower110 | NCT02409342 | 30.000 | 37.789 |
| IMpower133 | NCT02763579 | 19.332 | 24.647 |
| IMspire150 | NCT02908672 | 30.177 | 53.181 |
| INAVO120 | NCT04191499 | 40.200 | 52.800 |
| INo-VATEALL | NCT01564784 | 31.142 | 43.771 |
| JAVELIN 100 | NCT02603432. | 35.786 | 55.329 |
| JAVELIN 101 | NCT02684006 | 22.439 | 84.601 |
| JUPITER-02 | NCT03581786 | 25.250 | 41.600 |
| KATHERINE | NCT01772472 | 61.900 | 122.500 |
| KEYNoTE-006-PEMQ2IPI | NCT01866319 | 16.845 | 58.787 |
| KEYNoTE-006-PEMQ3IPI | NCT01866319 | 16.938 | 59.727 |
| KEYNoTE-024 | NCT02142738 | 17.540 | 59.320 |
| KEYNoTE-042 | NCT02220894 | 34.842 | 71.755 |
| KEYNoTE-045 | NCT02256436 | 18.347 | 66.665 |
| KEYNoTE-048 (P-C) | NCT02358031 | 41.744 | 49.828 |
| KEYNoTE-048 (PC-C) | NCT02358031 | 34.485 | 50.110 |
| KEYNoTE-177 | NCT02563002 | 58.400 | 82.870 |
| KEYNoTE-189 | NCT02578680 | 17.252 | 60.010 |
| KEYNoTE-407 | NCT02775435 | 16.800 | 58.300 |
| KEYNoTE-426 | NCT02853331 | 20.000 | 36.000 |
| KEYNoTE-522 | NCT03036488 | 46.875 | 82.995 |
| KEYNoTE-564 | NCT03142334 | 40.431 | 73.229 |
| KEYNoTE-671 | NCT03425643 | 49.294 | 60.607 |
| KEYNoTE-775 | NCT03517449 | 24.000 | 37.059 |
| KEYNoTE-826 | NCT03635567 | 28.100 | 60.600 |
| KEYNoTE-A18 | NCT04221945 | 28.250 | 41.050 |
| LATITUDE | NCT01715285 | 42.000 | 60.000 |
| MAGNITUDE | NCT03748641 | 28.331 | 46.549 |
| MAIA | NCT02252172 | 38.810 | 63.200 |
| MARIPOSA | NCT04487080 | 31.737 | 47.777 |
| METEOR | NCT01865747 | 18.300 | 26.670 |
| METRIC | NCT01245062 | 8.513 | 69.494 |
| MONALEESA-7 | NCT02278120 | 43.877 | 61.947 |
| monarchE | NCT03155997 | 57.262 | 68.786 |
| MPACT | NCT00844649 | 31.022 | 34.049 |
| MURANo | NCT02005471 | 27.200 | 54.000 |
| NAPOLI-1 (N-F) | NCT01494506 | 17.150 | 26.900 |
| NAPOLI-1 (NF-F) | NCT01494506 | 13.000 | 27.000 |
| NATALEE | NCT03701334 | 44.868 | 50.877 |
| NCT01343277 | NCT01343277 | 22.761 | 34.241 |
| NCT01539512 | NCT01539512 | 14.404 | 66.845 |
| NETTER-1 | NCT01578239 | 22.191 | 84.659 |
| NSABP B31 and NCCTG N9831 | NCT00004067 | 53.682 | 121.963 |
| OAK | NCT02008227 | 26.132 | 52.934 |
| OlympiAD | NCT02000622 | 26.786 | 35.579 |
| OPTiM | NCT00769704 | 50.000 | 52.000 |
| PACIFIC | NCT02125461 | 38.230 | 69.681 |
| PANoRAMA | NCT01023308 | 41.048 | 62.764 |
| POLO | NCT02184195 | 34.719 | 48.339 |
| POPLAR | NCT01903993 | 17.808 | 48.000 |
| POSEIDON | NCT03164616 | 44.122 | 73.124 |
| PRIMA | NCT00140582 | 51.519 | 135.474 |
| PROFILE 1014 | NCT01526928 | 32.494 | 65.622 |
| PROfound | NCT02987543 | 21.169 | 30.108 |
| PROLONG | NCT01039376 | 29.900 | 75.300 |
| PROSPER | NCT02003924 | 39.345 | 67.311 |
| PSMAfore (NCT04689828) | NCT04689828 | 29.700 | 39.500 |
| RADIANT-3 | NCT00510068 | 27.827 | 73.377 |
| RECORD-1 | NCT00410124 | 10.150 | 20.550 |
| RELAY | NCT02411448 | 34.276 | 77.421 |
| RUBY | NCT03981796 | 34.846 | 46.422 |
| SEQUOIA | NCT03336333 | 36.841 | 76.759 |
| SOPHIA | NCT02492711 | 37.393 | 57.266 |
| SPARTAN | NCT01946204 | 38.958 | 68.000 |
| SSGXVIII | NCT00116935 | 71.723 | 136.883 |
| S-TRAC | NCT00375674 | 97.865 | 108.460 |
| SUN 111 | NCT00428597 | 20.539 | 80.294 |
| TALAPRO-2 | NCT03395197 | 40.511 | 65.402 |
| TAX 324 | NCT00273546 | 74.185 | 106.667 |
| TITAN | NCT02489318 | 31.622 | 53.478 |
| TIVO-3 | NCT02627963 | 37.193 | 42.000 |
| TOPAZ-1 | NCT03875235 | 25.773 | 32.156 |
| TRANSFORM | NCT03575351 | 19.175 | 35.353 |
| TROPiCS-02 | NCT03901339 | 25.101 | 33.156 |
| VIALE-A | NCT02993523 | 28.136 | 42.425 |
| VISTA | NCT00111319 | 28.456 | 42.425 |
| ZUMA-7 | NCT03391466 | 35.246 | 50.000 |

# Table S7 The distribution of trial-specific τ values for EFS/PFS

| Trial | NCT | Initial τ/month | Updated τ/month |
| --- | --- | --- | --- |
| ACOSOG Z9001 | NCT00041197 | 48.000 | 72.300 |
| ADAURA | NCT02511106 | 32.062 | 64.564 |
| AETHERA | NCT01100502 | 25.500 | 84.300 |
| ALCANZA | NCT01578499 | 12.000 | 30.924 |
| ALEX | NCT02075840 | 25.000 | 45.190 |
| ALTA-1L | NCT02737501 | 15.000 | 26.735 |
| APHINITY | NCT01358877 | 47.833 | 71.345 |
| ARCHES | NCT02677896 | 20.042 | 52.83 |
| ASCENT | NCT02574455 | 9.290 | 14.700 |
| ATTRACTION-3 | NCT02569242 | 23.500 | 38.800 |
| BEACONCRC | NCT02928224 | 18.752 | 21.780 |
| BOLERO-2 | NCT00863655 | 15.200 | 17.800 |
| BREAK-3 | NCT01227889 | 7.430 | 46.100 |
| CANDOR | NCT03158688 | 20.000 | 28.686 |
| CASPIAN | NCT03043872 | 15.79 | 21.62 |
| CASSIOPEIA | NCT02541383 | 28.405 | 53.997 |
| CASTOR | NCT02136134 | 12.400 | 19.500 |
| CheckMate 816 | NCT02998528 | 41.300 | 58.400 |
| CheckMate 9LA | NCT03215706 | 32.900 | 73.500 |
| CheckMate025 | NCT01668784 | 24.275 | 41.389 |
| CheckMate057 | NCT01673867 | 22.586 | 27.097 |
| CheckMate214 | NCT02231749 | 27.484 | 37.951 |
| CheckMate227 | NCT02477826 | 36.568 | 67.084 |
| CheckMate238 | NCT02388906 | 24.936 | 52.308 |
| CHECKMATE-274 | NCT02632409 | 48.400 | 63.350 |
| CHECKMATE-648 (NC-C) | NCT03143153 | 26.200 | 50.100 |
| CHECKMATE-648 (NI-C) | NCT03143153 | 26.200 | 50.100 |
| CheckMate649 | NCT02872116 | 33.629 | 36.083 |
| CheckMate743 | NCT02899299 | 33.539 | 34.316 |
| CheckMate9ER | NCT03141177 | 22.608 | 37.349 |
| CLEAR | NCT02811861 | 28.166 | 32.980 |
| CLEOPATRA | NCT00567190 | 41.850 | 119.000 |
| CLL | NCT02242942 | 29.336 | 70.929 |
| CLL8 | NCT00281918 | 59.700 | 93.400 |
| coBRIM | NCT01689519 | 10.700 | 65.000 |
| COLUMBUS (ENB-EN) | NCT01909453 | 22.571 | 104.503 |
| COLUMBUS (ENB-VEM) | NCT01909453 | 21.101 | 101.705 |
| COMBI-AD | NCT01682083 | 48.000 | 71.371 |
| COMBI-d | NCT01584648 | 13.400 | 40.300 |
| COSMIC-311 | NCT03690388 | 4.312 | 12.900 |
| COU-AA-302 | NCT00887198 | 27.500 | 30.300 |
| CROWN | NCT03052608 | 26.085 | 65.999 |
| DESTINY-Breast03 | NCT03529110 | 28.712 | 53.472 |
| E1912 | NCT02048813 | 47.683 | 79.055 |
| ECHELON-1 | NCT01712490 | 43.000 | 80.107 |
| ECHELON-2 | NCT01777152 | 50.939 | 64.429 |
| ELEVATE-TN (A-O) | NCT02475681 | 39.500 | 85.301 |
| ELEVATE-TN (AO-O) | NCT02475681 | 39.300 | 85.301 |
| ELOQUENT-2 | NCT01239797 | 34.131 | 57.000 |
| EMPOWER-Lung1 | NCT03088540 | 16.597 | 31.319 |
| EMPOWER-Lung3 | NCT03409614 | 18.657 | 24.000 |
| ExteNET | NCT00878709 | 24.000 | 60.000 |
| GADOLIN | NCT01059630 | 40.833 | 55.000 |
| GALLIUM | NCT01332968 | 51.600 | 103.000 |
| GO29365 | NCT02257567 | 21.000 | 26.300 |
| GOG 240 | NCT00803062 | 29.800 | 51.100 |
| HER2CLIMB | NCT02614794 | 10.839 | 12.000 |
| ICARIA-MM | NCT02990338 | 15.380 | 38.916 |
| IKEMA | NCT03275285 | 24.670 | 41.983 |
| IMbrave150 | NCT03434379 | 12.562 | 23.273 |
| IMCgp100-202 | NCT03070392 | 16.389 | 26.695 |
| IMpassion130 | NCT02425891 | 22.410 | 33.007 |
| IMpower110 | NCT02409342 | 17.981 | 32.702 |
| IMspire150 | NCT02908672 | 29.000 | 49.447 |
| INAVO120 | NCT04191499 | 37.600 | 52.100 |
| INo-VATEALL | NCT01564784 | 16.000 | 19.154 |
| JAVELIN 100 | NCT02603432. | 26.531 | 47.077 |
| JAVELIN 101 | NCT02684006 | 19.426 | 77.651 |
| KATHERINE | NCT01772472 | 61.550 | 121.500 |
| KEYNoTE-006-PEMQ2IPI | NCT01866319 | 9.479 | 44.783 |
| KEYNoTE-006-PEMQ3IPI | NCT01866319 | 9.479 | 44.783 |
| KEYNoTE-024 | NCT02142738 | 14.272 | 47.275 |
| KEYNoTE-042 | NCT02220894 | 28.429 | 59.968 |
| KEYNoTE-045 | NCT02256436 | 15.646 | 60.217 |
| KEYNoTE-048 (P-C) | NCT02358031 | 32.000 | 47.969 |
| KEYNoTE-048 (PC-C) | NCT02358031 | 29.569 | 49.070 |
| KEYNoTE-054 | NCT02362594 | 23.000 | 72.700 |
| KEYNoTE-177 | NCT02563002 | 36.312 | 72.704 |
| KEYNoTE-189 | NCT02578680 | 17.942 | 40.105 |
| KEYNoTE-355 | NCT02819518 | 31.750 | 46.400 |
| KEYNoTE-407 | NCT02775435 | 15.500 | 57.000 |
| KEYNoTE-426 | NCT02853331 | 18.000 | 30.000 |
| KEYNoTE-522 | NCT03036488 | 24.424 | 81.712 |
| KEYNoTE-564 | NCT03142334 | 37.700 | 69.187 |
| KEYNoTE-671 | NCT03425643 | 44.550 | 57.300 |
| KEYNoTE-716 | NCT03553836 | 31.300 | 61.191 |
| KEYNoTE-775 | NCT03517449 | 23.535 | 34.100 |
| KEYNoTE-826 | NCT03635567 | 24.600 | 42.600 |
| KEYNoTE-A18 | NCT04221945 | 26.000 | 37.300 |
| MAGNITUDE | NCT03748641 | 27.599 | 34.828 |
| MAIA | NCT02252172 | 38.574 | 55.938 |
| METRIC | NCT01245062 | 7.110 | 20.285 |
| MONARCH 2 | NCT02107703 | 24.000 | 45.000 |
| MONARCH 3 | NCT02246621 | 21.799 | 95.432 |
| monarchE | NCT03155997 | 28.341 | 46.350 |
| MURANo | NCT02005471 | 33.000 | 51.000 |
| NAPOLI-1 (N-F) | NCT01494506 | 8.050 | 15.000 |
| NAPOLI-1 (NF-F) | NCT01494506 | 12.000 | 17.400 |
| NATALEE | NCT03701334 | 43.372 | 49.789 |
| NCT01539512 | NCT01539512 | 13.595 | 14.096 |
| NSABP B31 and NCCTG N9831 | NCT00004067 | 54.534 | 123.799 |
| PACIFIC | NCT02125461 | 21.000 | 65.833 |
| PALOMA3 | NCT01942135 | 10.829 | 20.032 |
| PAOLA-1 | NCT02477644 | 34.631 | 49.084 |
| PARAMOUNT | NCT00789373 | 10.050 | 25.582 |
| POLLUX | NCT02076009 | 15.889 | 49.547 |
| PRIMA | NCT00140582 | 51.359 | 133.770 |
| PROLONG | NCT01039376 | 28.300 | 75.100 |
| REACH3 | NCT03112603 | 26.633 | 38.762 |
| RECORD-1 | NCT00410124 | 6.230 | 8.805 |
| RELATIVITY-047 | NCT03470922 | 28.767 | 72.425 |
| RELAY | NCT02411448 | 33.000 | 78.737 |
| SEQUOIA | NCT03336333 | 36.318 | 68.449 |
| SOLO-1 | NCT01844986 | 47.584 | 65.807 |
| SOPHIA | NCT02492711 | 17.679 | 51.561 |
| SSGXVIII | NCT00116935 | 67.307 | 131.780 |
| TALAPRO-2 | NCT03395197 | 40.730 | 64.337 |
| TAX 324 | NCT00273546 | 70.797 | 109.729 |
| TRANSFORM | NCT03575351 | 17.897 | 33.148 |
| ZUMA-7 | NCT03391466 | 24.000 | 46.000 |

# Table S8 Overall and Subgroup Meta-analysis of RR (Ratio of updated RMST ratio to initial RMST ratio) for OS

| Variable | Subgroup | No. of studies | RR (95% CI) | p-value | I^2^ |
| --- | --- | --- | --- | --- | --- |
| Overall | All Studies | 150 | 1.023 (1.015–1.031) | 0.000 | 15.5 |
| Number of Patients | Large(>600) | 60 | 1.022 (1.012–1.032) | 0.000 | 33.7 |
| Number of Patients | Medium (100-600) | 89 | 1.029 (1.014–1.044) | 0.000 | 0 |
| FDA Approve Year | Pre-2015 | 29 | 1.031 (1.015–1.046) | 0.000 | 0 |
| FDA Approve Year | 2016–2020 | 75 | 1.027 (1.015–1.038) | 0.000 | 11.9 |
| FDA Approve Year | Post-2020 | 46 | 1.010 (0.999–1.020) | 0.076 | 8.3 |
| 5-year survival of indication | Intermediate (30%-69%) | 85 | 1.028 (1.017–1.039) | 0.000 | 0 |
| 5-year survival of indication | High survival (70%-1) | 23 | 1.011 (0.999–1.023) | 0.076 | 47.6 |
| 5-year survival of indication | Low survival (<30%) | 42 | 1.040 (1.021–1.059) | 0.000 | 0 |
| PH Assumption | PH | 110 | 1.020 (1.012–1.028) | 0.000 | 16.9 |
| PH Assumption | Non-PH | 40 | 1.036 (1.017–1.055) | 0.000 | 0 |
| Treatment Line | First-line | 89 | 1.020 (1.011–1.029) | 0.000 | 20.8 |
| Treatment Line | Subsequent Line | 61 | 1.036 (1.019–1.052) | 0.000 | 0 |
| Treatement comparisons | Targeted VS. Supportive care/Placebo | 10 | 1.013 (0.987–1.040) | 0.337 | 0 |
| Treatement comparisons | Targeted VS. Chemotherapy | 9 | 1.032 (0.960–1.111) | 0.391 | 0 |
| Treatement comparisons | Targeted-Chemotherapy VS. Chemotherapy | 11 | 1.031 (1.010–1.052) | 0.004 | 0 |
| Treatement comparisons | Targeted VS. Targeted | 16 | 1.030 (1.006–1.053) | 0.012 | 0 |
| Treatement comparisons | Others | 27 | 1.027 (1.008–1.047) | 0.005 | 27.6 |
| Treatement comparisons | Hormonal VS. Hormonal | 6 | 1.041 (1.015–1.067) | 0.002 | 0 |
| Treatement comparisons | Immunotherapy VS. Chemotherapy | 13 | 1.075 (1.022–1.131) | 0.005 | 0 |
| Treatement comparisons | Immuno-Chemotherapy VS. Chemotherapy | 18 | 1.037 (1.011–1.063) | 0.005 | 0.2 |
| Treatement comparisons | Immuno-Targeted VS. Targeted | 8 | 1.034 (0.999–1.069) | 0.058 | 0 |
| Treatement comparisons | Immunotherapy VS. Targeted | 4 | 1.061 (0.993–1.134) | 0.078 | 0 |
| Treatement comparisons | Immuno-Targeted VS. Immuno-Chemotherapy | 4 | 1.016 (0.984–1.049) | 0.330 | 0 |
| Treatement comparisons | Chemotherapy VS. Chemotherapy | 6 | 1.018 (0.931–1.114) | 0.693 | 0 |
| Treatement comparisons | Immunotherapy VS. Immunotherapy | 4 | 1.077 (0.987–1.175) | 0.098 | 0 |
| Treatement comparisons | Immunotherapy VS. Supportive care/Placebo | 8 | 0.992 (0.959–1.025) | 0.613 | 31.3 |
| Treatement comparisons | Targeted-Hormonal VS. Hormonal | 6 | 0.998 (0.990–1.007) | 0.671 | 0 |
| Blinding | Yes | 51 | 1.014 (1.006–1.022) | 0.001 | 0 |
| Blinding | No | 99 | 1.023 (1.012–1.034) | 0.000 | 18 |
| Phase | 3 | 146 | 1.023 (1.015–1.031) | 0.000 | 15.5 |
| Phase | 2 | 4 | 1.094 (0.929–1.288) | 0.280 | 0 |
| Crossover | Yes | 59 | 1.017 (1.008–1.026) | 0.000 | 0 |
| Crossover | No | 91 | 1.019 (1.009–1.028) | 0.000 | 12.6 |
| Disease | Others | 29 | 1.024 (1.004–1.043) | 0.016 | 0 |
| Disease | Leukemia | 18 | 1.014 (0.992–1.037) | 0.216 | 0 |
| Disease | Lung Cancer | 25 | 1.049 (1.022–1.076) | 0.000 | 0 |
| Disease | Breast Cancer | 18 | 1.003 (0.996–1.010) | 0.398 | 2.4 |
| Disease | Prostate Cancer | 12 | 1.037 (1.017–1.058) | 0.000 | 0 |
| Disease | Lymphoma | 8 | 0.995 (0.953–1.038) | 0.805 | 32.9 |
| Disease | Colorectal Cancer | 7 | 1.055 (0.988–1.126) | 0.109 | 0 |
| Disease | Head and Neck Cancer | 5 | 1.047 (0.959–1.143) | 0.310 | 0 |
| Disease | Kidney Cancer | 12 | 1.023 (0.998–1.048) | 0.074 | 0 |
| Disease | Multiple Myeloma | 6 | 1.096 (1.003–1.198) | 0.043 | 81.2 |
| Disease | Melanoma | 10 | 1.056 (1.000–1.115) | 0.050 | 0 |
| Metastatic | No | 36 | 1.012 (1.003–1.021) | 0.012 | 27.7 |
| Metastatic | Yes | 114 | 1.036 (1.025–1.048) | 0.000 | 0 |
| Update time duration | Medium (12-24 months) | 41 | 1.032 (1.016–1.048) | 0.000 | 0 |
| Update time duration | Short (<12 months) | 47 | 1.001 (0.993–1.009) | 0.840 | 0 |
| Update time duration | Long (>24 months) | 62 | 1.027 (1.016–1.039) | 0.000 | 18.8 |
| Information fraction of initial OS | Immature (<50%) | 58 | 1.018 (1.009–1.028) | 0.000 | 29.4 |
| Information fraction of initial OS | Moderate (50%-75%) | 35 | 1.047 (1.024–1.071) | 0.000 | 0 |
| Information fraction of initial OS | Mature (>75%) | 57 | 1.027 (1.010–1.043) | 0.002 | 0 |

Note, The grouping criteria for each subgroup are described in Table S2; Subgroups with fewer than two studies were excluded from quantitative analyses; RMST ratio represents comparisons of the experimental group versus the control group; OS, overall survival

# Table S9 Overall and Subgroup Meta-analysis of RR (Ratio of updated RMST ratio to initial RMST ratio) for PFS/EFS

| Variable | Subgroup | No. of studies | RR (95% CI) | p-value | I^2^ |
| --- | --- | --- | --- | --- | --- |
| Overall | All Studies | 120 | 1.099 (1.077–1.121) | 0.000 | 69.2 |
| Number of Patients | Large(>600) | 53 | 1.026 (1.018–1.034) | 0.000 | 46.2 |
| Number of Patients | Medium (100-600) | 66 | 1.154 (1.126–1.184) | 0.000 | 18.8 |
| FDA Approve Year | Pre-2015 | 20 | 1.035 (1.006–1.065) | 0.019 | 0.0 |
| FDA Approve Year | 2016–2020 | 62 | 1.040 (1.029–1.051) | 0.000 | 63.7 |
| FDA Approve Year | Post-2020 | 38 | 1.032 (1.020–1.045) | 0.000 | 43.3 |
| 5-year survival of indication | Intermediate (30%-69%) | 76 | 1.041 (1.027–1.055) | 0.000 | 23.3 |
| 5-year survival of indication | Low survival (<30%) | 27 | 1.116 (1.077–1.157) | 0.000 | 10.3 |
| 5-year survival of indication | High survival (70%-1) | 17 | 1.029 (1.020–1.039) | 0.000 | 85.1 |
| PH Assumption | PH | 62 | 1.039 (1.024–1.054) | 0.000 | 32.5 |
| PH Assumption | Non-PH | 58 | 1.036 (1.027–1.045) | 0.000 | 64.9 |
| Treatment Line | First-line | 74 | 1.036 (1.027–1.044) | 0.000 | 61.8 |
| Treatment Line | Subsequent Line | 46 | 1.041 (1.022–1.060) | 0.000 | 27.0 |
| Treatement comparisons | Targeted VS. Supportive care/Placebo | 10 | 1.013 (0.992–1.034) | 0.224 | 44.5 |
| Treatement comparisons | Targeted VS. Chemotherapy | 5 | 1.104 (0.953–1.280) | 0.187 | 0.0 |
| Treatement comparisons | Targeted VS. Targeted | 14 | 1.087 (1.043–1.134) | 0.000 | 38.7 |
| Treatement comparisons | Others | 28 | 1.028 (1.015–1.041) | 0.000 | 38.9 |
| Treatement comparisons | Immunotherapy VS. Chemotherapy | 11 | 1.177 (1.093–1.268) | 0.000 | 0.0 |
| Treatement comparisons | Targeted-Hormonal VS. Hormonal | 9 | 1.028 (1.015–1.042) | 0.000 | 84.5 |
| Treatement comparisons | Immuno-Targeted VS. Targeted | 8 | 1.104 (1.041–1.171) | 0.001 | 0.0 |
| Treatement comparisons | Immuno-Chemotherapy VS. Chemotherapy | 12 | 1.065 (1.023–1.109) | 0.002 | 24.9 |
| Treatement comparisons | Immunotherapy VS. Immunotherapy | 4 | 1.127 (1.027–1.236) | 0.012 | 49.7 |
| Treatement comparisons | Immunotherapy VS. Supportive care/Placebo | 11 | 1.074 (1.032–1.118) | 0.000 | 0.0 |
| Treatement comparisons | Immuno-Targeted VS. Immuno-Chemotherapy | 4 | 1.237 (1.164–1.314) | 0.000 | 64.3 |
| Treatement comparisons | Targeted-Chemotherapy VS. Chemotherapy | 4 | 1.018 (0.986–1.051) | 0.264 | 0.0 |
| Blinding | Yes | 44 | 1.024 (1.013–1.035) | 0.000 | 24.3 |
| Blinding | No | 76 | 1.048 (1.037–1.059) | 0.000 | 60.0 |
| Disease | Others | 29 | 1.062 (1.028–1.096) | 0.000 | 7.9 |
| Disease | Lung Cancer | 20 | 1.146 (1.097–1.198) | 0.000 | 19.8 |
| Disease | Lymphoma | 11 | 1.023 (0.982–1.065) | 0.281 | 0.0 |
| Disease | Breast Cancer | 19 | 1.018 (1.010–1.027) | 0.000 | 0.0 |
| Disease | MelaNoma | 15 | 1.098 (1.051–1.146) | 0.000 | 4.6 |
| Disease | Multiple Myeloma | 8 | 1.132 (1.088–1.178) | 0.000 | 80.0 |
| Disease | Kidney Cancer | 8 | 1.076 (1.021–1.134) | 0.006 | 0.0 |
| Disease | Leukemia | 10 | 1.185 (1.135–1.238) | 0.000 | 54.6 |
| Metastatic | No | 27 | 1.030 (1.021–1.040) | 0.000 | 77.5 |
| Metastatic | Yes | 93 | 1.052 (1.037–1.067) | 0.000 | 30.1 |
| Update time duration | Long (>24 months) | 49 | 1.051 (1.037–1.064) | 0.000 | 65.6 |
| Update time duration | Medium (12-24 months) | 41 | 1.029 (1.018–1.041) | 0.000 | 53.4 |
| Update time duration | Short (<12 months) | 30 | 1.029 (1.013–1.045) | 0.000 | 0.0 |
| Information fraction of initial PFS/EFS | Immature (<50%) | 11 | 1.059 (1.026–1.093) | 0.000 | 35.8 |
| Information fraction of initial PFS/EFS | Moderate (50%-75%) | 47 | 1.035 (1.024–1.045) | 0.000 | 72.7 |
| Information fraction of initial PFS/EFS | Mature (>75%) | 62 | 1.036 (1.024–1.048) | 0.000 | 9.3 |

Note, The grouping criteria for each subgroup are described in Table S2; Subgroups with fewer than two studies were excluded from quantitative analyses; RMST ratio represents comparisons of the experimental group versus the control group; PFS, and EFS refer to progression-free survival, and event-free (including disease-free or relapse-free) survival, respectively

# Table S10 Univariable meta-regression results of overall survival (OS) by individual covariates

| Variable | Regression Coefficient | 95% CI Lower Limit | 95% CI Upper Limit | p‑Value |
| --- | --- | --- | --- | --- |
| Information fraction of initial OS | 0.063 | 0.036 | 0.091 | 0.000 |
| Phase | -0.079 | -0.242 | 0.084 | 0.344 |
| Crossover | 0.008 | -0.002 | 0.019 | 0.131 |
| Initial data cutoff time | -0.001 | -0.001 | 0.000 | 0.000 |
| Updated data cutoff time | 0.000 | 0.000 | 0.000 | 0.437 |
| Follow‑up duration | 0.000 | 0.000 | 0.001 | 0.009 |
| Initial censoring rates (experimental group) | -0.071 | -0.102 | -0.040 | 0.000 |
| Initial censoring rates (control group) | -0.068 | -0.094 | -0.042 | 0.000 |
| Updated censoring rates (experimental group) | -0.069 | -0.092 | -0.045 | 0.000 |
| Updated censoring rates (control group) | -0.070 | -0.091 | -0.050 | 0.000 |
| Proportional‑hazards status | 0.026 | 0.007 | 0.045 | 0.007 |
| Treatment Line | 0.026 | 0.009 | 0.043 | 0.002 |
| Trial sample size | 0.019 | 0.004 | 0.035 | 0.014 |
| Blinding status | 0.004 | -0.006 | 0.015 | 0.402 |
| 5-Year Survival Rate | 0.061 | -0.061 | 0.183 | 0.329 |
| Approve Year | -0.001 | -0.003 | 0.000 | 0.010 |
| Metastatic setting | 0.031 | 0.018 | 0.043 | 0.000 |

Note, The grouping criteria for each subgroup are described in Table S1

# Table S11 Univariable meta-regression results of PFS/EFS by individual covariates

| Variable | Regression Coefficient | 95% CI Lower Limit | 95% CI Upper Limit | p‑Value |
| --- | --- | --- | --- | --- |
| Information fraction of initial PFS/EFS | -0.053 | -0.108 | 0.003 | 0.062 |
| Phase | 0.049 | -0.642 | 0.740 | 0.889 |
| Initial data cutoff time | -0.001 | -0.002 | -0.001 | 0.000 |
| Updated data cutoff time | 0.000 | -0.001 | 0.000 | 0.298 |
| Follow‑up duration | 0.000 | 0.000 | 0.001 | 0.082 |
| Initial censoring rates (experimental group) | -0.123 | -0.161 | -0.085 | 0.000 |
| Initial censoring rates (control group) | -0.145 | -0.176 | -0.113 | 0.000 |
| Updated censoring rates (experimental group) | -0.133 | -0.165 | -0.100 | 0.000 |
| Updated censoring rates (control group) | -0.144 | -0.173 | -0.115 | 0.000 |
| Proportional‑hazards status | 0.003 | -0.014 | 0.020 | 0.721 |
| Treatment Line | 0.005 | -0.015 | 0.025 | 0.621 |
| Trial sample size | 0.118 | 0.092 | 0.145 | 0.000 |
| Blinding status | -0.023 | -0.038 | -0.008 | 0.003 |
| 5-Year Survival Rate | -0.049 | -0.082 | -0.017 | 0.003 |
| Approve Year | 0.001 | -0.001 | 0.003 | 0.300 |
| Metastatic setting | 0.021 | 0.004 | 0.037 | 0.015 |

Note, The grouping criteria for each subgroup are described in Table S1

# Table S12 Multivariable meta-regression results of OS

| Variable | Regression Coefficient | 95% CI Lower Limit | 95% CI Upper Limit | p Value |
| --- | --- | --- | --- | --- |
| Intercept | 2.339 | -0.884 | 5.562 | 0.155 |
| **Updated censoring rates (control group)** | **-0.266** | **-0.385** | **-0.147** | **0.000** |
| **Initial censoring rates (control group)** | **0.167** | **0.027** | **0.308** | **0.019** |
| Updated censoring rates (treatment group) | 0.119 | -0.005 | 0.243 | 0.061 |
| Approve Year | -0.001 | -0.003 | 0.000 | 0.165 |
| Proportional-hazards status | 0.014 | -0.009 | 0.037 | 0.223 |
| Metastatic setting | -0.013 | -0.036 | 0.011 | 0.301 |
| Initial data cutoff time | 0.000 | -0.001 | 0.000 | 0.343 |
| Initial censoring rates (treatment group) | -0.064 | -0.214 | 0.087 | 0.406 |
| Follow-up duration | 0.000 | 0.000 | 0.000 | 0.500 |
| Treatment Line | -0.006 | -0.029 | 0.016 | 0.580 |
| Trial sample size | 0.165 | -0.519 | 0.850 | 0.636 |
| Information fraction of initial OS | 0.004 | -0.041 | 0.048 | 0.866 |

Note: All variables included in the regression model had variance inflation factors (VIFs) less than 5, indicating no multicollinearity; The model achieved an R^2^ of 1. Given the low baseline between-study heterogeneity for OS (I^2^=15.5%), the residual τ^2^ after covariate adjustment was near zero, yielding a pseudo-R^2^ of 100%. This reflects the limited residual heterogeneity rather than model overfitting; the pre-specified rule of ≥10 studies per covariate was strictly applied to guard against the latter. The grouping criteria for each subgroup are provided in Table S1.

# Table S13 Multivariable meta-regression results of PFS/EFS

| Variable | Regression Coefficient | 95% CI Lower Limit | 95% CI Upper Limit | p Value |
| --- | --- | --- | --- | --- |
| Intercept | 0.0751 | -0.0250 | 0.1753 | 0.1420 |
| **Updated censoring rates (treatment group)** | **0.3646** | **0.1936** | **0.5357** | **0.0000** |
| **Updated censoring rates (control group)** | **-0.5942** | **-0.7959** | **-0.3925** | **0.0000** |
| **Initial censoring rates (control group)** | **0.2394** | **0.0730** | **0.4058** | **0.0048** |
| **Initial data cutoff time** | **-0.0012** | **-0.0023** | **0.0000** | **0.0470** |
| Trial sample size (group = Medium) | 0.0315 | -0.0054 | 0.0683 | 0.0941 |
| Initial censoring rates (treatment group) | -0.0900 | -0.2181 | 0.0382 | 0.1690 |
| Metastatic setting | -0.0286 | -0.0713 | 0.0141 | 0.1890 |
| Blinding status | 0.0109 | -0.0092 | 0.0310 | 0.2870 |
| Follow‑up duration | 0.0004 | -0.0004 | 0.0012 | 0.3290 |
| Information fraction of initial PFS/EFS | 0.0341 | -0.0377 | 0.1058 | 0.3520 |
| Trial sample size | -0.0775 | -0.7696 | 0.6147 | 0.8260 |
| 5‑Year Survival Rate | -0.0040 | -0.0992 | 0.0912 | 0.9340 |

Note: All variables included in the regression model had variance inflation factors (VIFs) less than 5, indicating no multicollinearity; The model achieved an R^2^ of 0.74; The pseudo-R^2^ of 74% indicates that the included covariates explained approximately three-quarters of the between-study variance (τ^2^) for PFS/EFS (baseline I^2^=69.2%, residual I^2^=21.7%). The grouping criteria for each subgroup are provided in Table S1

# Result S1 Detailed results for multivariable meta-regression analyses

For overall survival (OS), both a higher initial censoring rate (β = 0.167, p = 0.019) and a lower updated censoring rate (β = –0.266, p < 0.001) in the control arm were independently associated with a larger RR. Clinically, this means that when the control group had more censored patients initially but fewer after data maturity, the updated analysis revealed a stronger relative survival advantage for the experimental group. Other study‑level factors were not significant.

For progression‑free or event‑free survival (PFS/EFS), censoring‑related variables dominated again. Greater updated censoring in the treatment arm (β = 0.365, p < 0.001), lower updated censoring in the control arm (β = –0.594, p < 0.001), and higher initial censoring in the control arm (β = 0.239, p = 0.005) were significantly correlated with a higher RR. A shorter initial data‑cutoff time (β = –0.0012, p = 0.047) was also associated with a greater RR. These results suggest that when the initial analyses were based on less mature follow‑up but later updates captured more complete control‑arm events, the experimental group exhibited a stronger relative improvement in PFS/EFS.

# Reference

1. Soon YY, Marschner IC, Schou M, et al. Challenges of estimating treatment effects after a positive interim analysis. *European Journal of Cancer*. 2024;209:114230. doi:10.1016/j.ejca.2024.114230

2. Siegel RL, Giaquinto AN, Jemal A. Cancer statistics, 2024. *CA Cancer J Clin*. 2024;74(1):12-49. doi:10.3322/caac.21820

3. Kearns B, Stevenson MD, Triantafyllopoulos K, Manca A. Comparing current and emerging practice models for the extrapolation of survival data: a simulation study and case-study. *BMC Med Res Methodol*. 2021;21(1):263. doi:10.1186/s12874-021-01460-1
